# Low- and high-dose post-transplant cyclophosphamide attenuates graft-versus-host disease with distinct effects on PD-1^+^ T cell subsets

**DOI:** 10.1042/CS20257272

**Published:** 2025-10-28

**Authors:** Chloe Sligar, Miles J. Jacobs, Amal Elhage, Ronald Sluyter, Debbie Watson

**Affiliations:** 1 Molecular Horizons and School of Science, University of Wollongong, Wollongong, NSW, 2522, Australia

**Keywords:** humanised mice, post-transplant cyclophosphamide, T cell exhaustion, xenogeneic graft-versus-host disease

## Abstract

Allogeneic haematopoietic stem cell transplantation (alloHSCT) is a curative treatment for haematological malignancies. AlloHSCT aims to generate graft-versus-leukaemia immunity, where donor T cells eliminate residual malignant cells. However, graft-versus-host disease (GVHD), where donor T cells attack recipient tissues, is a common and often fatal side effect. Post-transplant cyclophosphamide (PTCy) can reduce GVHD, but the cellular mechanisms through which this occurs are not fully understood, and high doses may be associated with toxicity. This study aimed to determine whether lower doses of PTCy can reduce GVHD and to examine the effects of PTCy doses on human (h) immune cell subsets, T cell exhaustion, and histological GVHD in a humanised mouse model. NOD-*scid*-IL2Rγ^null^ mice were injected with 2 × 10^7^ human peripheral blood mononuclear cells on day 0, cyclophosphamide (10, 25 or 33 mg/kg) or control diluent on days 3 and 4 and monitored for GVHD development at early and late time points. Low-dose PTCy (10 mg/kg) abrogated clinical signs of GVHD with comparable efficacy to high-dose PTCy (33 mg/kg), delaying GVHD onset and prolonging mouse survival. Proportions of hPD-1^+^ hCD4^+^ and hPD-1^+^hCD8^+^ T cells were increased with low-dose PTCy but not higher doses, while hPD-1^+^ hTreg proportions were increased by all PTCy doses. Exhausted hPD-1^+^hLAG3^+^hCD8^+^ T cell proportions were increased with high-dose PTCy, but not lower doses. This study indicates that low-dose PTCy reduces GVHD with similar efficacy to that of high-dose PTCy, but this appears to be associated with differing cellular mechanisms of action.

## Introduction

Allogeneic haematopoietic stem cell transplantation (alloHSCT) is a curative treatment for haematological malignancies, including leukaemia, that are refractory to chemotherapy and radiotherapy [1]. Following myeloablation, alloHSCT aims to generate graft-versus-leukaemia (GVL) immunity, where alloreactive donor T cells in the graft eliminate residual host malignant cells [2]. Despite its effectiveness, this treatment is frequently associated with graft-versus-host disease (GVHD), where alloreactive donor T cells also recognise the healthy tissues of the transplant recipient as foreign and mount a destructive inflammatory immune response [3]. GVHD results in severe damage to multiple organs, including the skin, liver, gastrointestinal tract and lungs [3–5]. One therapeutic strategy for the prevention of GVHD is the use of post-transplant cyclophosphamide (PTCy). While PTCy reduces GVHD occurrence and severity, this disease still develops in 40–50% of patients [6,7], and the cellular mechanisms of action by which PTCy reduces the disease are not fully understood.

In alloHSCT, PTCy is commonly delivered on days 3 and 4 post-transplant at a high dose (≥50 mg/kg), where it reduces GVHD [8–10]. Although inexpensive and effective in most cases, the administration of PTCy has been associated with impaired immune reconstitution [11–13] and toxicity [14]. Common toxicities associated with high doses of cyclophosphamide include cardiotoxicity [15], liver toxicity [16], gonadal and ovarian failure and haemorrhagic cystitis and fibrosis in the bladder [14]. Furthermore, cyclophosphamide is carcinogenic and can lead to bladder cancer, secondary acute leukaemia and kidney cancer [17,18], with higher cyclophosphamide doses associated with a greater incidence of these adverse events and increased patient mortality [16]. As such, the efficacy of low-dose PTCy following alloHSCT is now being explored.

In allogeneic mouse models, one study by Wachsmuth and colleagues found lower doses of PTCy (10 mg/kg and 25 mg/kg) reduced GVHD, with the latter being superior in reducing GVHD mortality, decreasing alloreactive CD4^+^ and CD8^+^ T cells and increasing donor regulatory T cells (Tregs) compared with both lower (10 mg/kg) and higher (50–200 mg/kg) PTCy doses [19]. Another study by Wang and colleagues found that low-dose PTCy (10 mg/kg) was able to mitigate GVHD, indicating a role for donor Treg reconstitution in this outcome [20].

Clinically, a low dose of PTCy (14.5 mg/kg) on days 3 and 4 post-transplant in combination with anti-thymocyte globulin significantly reduced the cumulative incidence of severe acute GVHD compared with anti-thymocyte globulin alone; however, 30% of these patients still developed moderate to severe chronic GVHD [21]. More recently, another clinical study involving elderly patients undergoing haploidentical alloHSCT revealed that PTCy at a lower dose (40 mg/kg) was found to have a similar efficacy to that of a high dose (50 mg/kg); however, 32% of patients administered this lower dose still developed GVHD, with 52% achieving GVHD-free, relapse-free survival at two years post-transplant [22].

Despite being used clinically for over a decade, the precise mechanism by which PTCy at high or low doses reduces GVHD and impacts alloreactive donor T cells remains unclear. Some proposed mechanisms of action include the depletion of proliferating effector T cells [23], the induction of effector T cell dysfunction or exhaustion [19], or the enhancement of Treg survival and function [24]. T cell exhaustion refers to a dysfunctional state induced by chronic antigen exposure, characterised by the gradual decline of T cell effector functions and self-renewal capacity [25,26]. PD-1 (or programmed cell death protein 1) is an inhibitory receptor present on activated T cells and plays a key role in modulating immune responses [27,28]. LAG3 (or lymphocyte activation gene 3) is expressed on multiple T cell subsets and is involved in T cell homeostasis, activation and proliferation [29]. T cells that co-express both PD-1 and LAG3 are considered exhausted, resulting in loss of effector functions and cell death due to overstimulation [30]. While exhausted effector T cells may be beneficial in the context of GVHD, the opposite may be true for generating GVL immunity. In an allogeneic mouse model of GVL immunity, T cell exhaustion was found to contribute to reduced GVL responses, which could be overcome via PD-1 blockade, but not via blockade of other exhaustion-associated receptors, including LAG3 [31]. Moreover, an association between CD4^+^ and CD8^+^ T cell exhaustion and relapse has been identified in patients with acute myeloid leukaemia [32] and B-cell acute lymphoblastic leukaemia [33]. As such, the impact of PTCy on T cell exhaustion warrants further investigation.

An imbalance of effector T cells and Tregs is associated with the development of GVHD following alloHSCT [34]. Tregs protect against GVHD through the secretion of regulatory cytokines, including interleukin (IL)-10, IL-35 and transforming growth factor beta [35,36], and through the overexpression of immune checkpoint inhibitors such as PD-1 [37]. In allogeneic mouse models, PD-1-deficient Tregs showed increased apoptosis, reducing Treg numbers and worsening GVHD [38]. This study also showed that a single 50 mg/kg dose of PTCy on day 3 post-transplant rescued PD-1 deficient Tregs from apoptosis, re-establishing the balance between regulatory and effector T cells through depletion of alloreactive T cells while sparing Tregs that are resistant to PTCy effects due to the expression of aldehyde dehydrogenase [6].

This study aimed to determine whether lower doses of PTCy (10 mg/kg and 25 mg/kg) are equally as efficacious as high-dose PTCy (33 mg/kg) in limiting GVHD in a preclinical humanised mouse model. This study also aimed to explore the cellular mechanisms of action by which different PTCy doses reduce GVHD by examining the impact of PTCy at each dose on human (h) immune cell subsets, with a focus on markers of T cell exhaustion at early and late disease time points in this preclinical model.

## Methods

### Mice

Approval for experiments involving mice was obtained from the University of Wollongong (Wollongong, Australia) Animal Ethics Committee (AE 20/01). Female, 5–7-week-old NOD-*scid*-IL2Rγ^null^ (NSG) (*n*=60 total; *n*=10 per treatment group for the long-term model and *n*=5 per treatment group for the short-term model) mice were obtained from Australian BioResources (Moss Vale, Australia) and housed in the Molecular Horizons Animal Research Facility, University of Wollongong. Mice, each from different treatment groups, were housed in individually ventilated cages (Tecniplast, Buggiate, Italy) under a 12 h light/12 h dark cycle, with four mice per cage. In alignment with the 3Rs, female mice were used to allow for group co-housing, as male mice are prone to aggression when co-housed [39]. Mice were provided with enrichments, irradiated mouse chow (Specialty Feeds, Glen Forrest, Australia) and autoclaved water *ad libitum*. Mice were acclimatised for two weeks before commencing experiments.

### Human peripheral blood mononuclear cell isolation

Approval for experiments involving human blood was obtained from the University of Wollongong Human Ethics Committee (HE 12/290) and with consent from healthy human donors (two females and four males, aged 21–30 years). Human peripheral blood mononuclear cells (hPBMCs) were isolated by density gradient centrifugation (562× ***g***, 30 min; break disengaged) using Ficoll-Paque PLUS (GE Healthcare, Uppsala, Sweden). hPBMCs were washed twice with sterile Dulbecco’s phosphate buffered saline (PBS) (Thermo Fisher Scientific, Waltham, U.S.A.) (440× ***g***, 10 min) and resuspended in PBS at 1 × 10^8^ cells/ml for injection into mice.

### Humanised mouse model of GVHD

NSG mice were then injected intraperitoneally (i.p.) with 2 × 10^7^ hPBMCs (day 0) from different donors (*n*=2–6 donors per experiment, described in detail in corresponding figure legends) using a 27-gauge × 12 mm needle and 1 ml syringe. Cyclophosphamide (40 mg/ml; Sigma Aldrich, St Louis, U.S.A.) was diluted to 6.6, 5 or 2 mg/ml with PBS and stored for up to 48 h at 4°C prior to injection. On days 3 and 4, mice were injected i.p. with either cyclophosphamide (33 mg/kg, 25 mg/kg or 10 mg/kg) or PBS using a 30-gauge × 8 mm insulin syringe. Mice were monitored and scored in a blinded fashion at least thrice weekly until endpoint as described [40], with mice euthanised by slow-fill CO_2_ at day 28 (short-term model), day 70 (long-term model) or ethical endpoint. Organs (spleen, liver, lung, ear and skin) were collected into PBS, RPMI-1640 medium (Thermo Fisher Scientific) and/or 10% neutral buffered formalin (Sigma Aldrich) as indicated.

### Histology

Livers, lungs, skin and ears collected from euthanised mice were fixed overnight in 10% neutral buffered formalin. Tissues were processed in an ASP200S tissue processor (Leica, Buffalo Grove, U.S.A.) and embedded in paraffin wax before being sectioned (3–5 μm) using an RM2255 microtome (Leica) and stained with haematoxylin and eosin (POCD, Artarmon, Australia) (Table 1). Histopathology was assessed using a DM750 inverted light microscope (Leica) with the 20X objective and Leica Application Suite software version 4.7 (Leica). Liver, skin and ear histopathology were assessed in a blinded manner using a standardised grading system (grades 0–4) as described [41]. Lung histopathology was assessed in a blinded manner by area measurements of open alveolar space using Fiji [42] and quantified as a percentage of the total measured lung area.

**Table 1:** Haematoxylin and eosin staining protocol

Step	Reagent	Time (sec)
1	Xylene substitute	150
2	100% ethanol	100
3	Water	120
4	Haematoxylin	100
5	Water	120
6	Acid alcohol (1% HCl in 100% ethanol)	1^1^
7	Water	60
8	Scott’s bluing reagent (45 mM NaHCO_3_, 166 mM MgSO_4_)	60
9	Water	60
10	Eosin	20
11	100% ethanol	105
12	Xylene substitute	70

1 Slides were dipped once in acid alcohol.

### Immunophenotyping

Cells were isolated from spleens and livers as described [43] before live/dead staining with Zombie near infrared (NIR) dye (BioLegend, San Diego, U.S.A.). Cells were then incubated with fluorochrome-conjugated monoclonal antibodies (Table 2) in PBS containing 2% foetal calf serum for 10 min on ice, protected from light. Cells were washed in PBS (300× ***g***, 5 min) and resuspended in 250 μl PBS for flow cytometric data acquisition. Data were obtained using an LSRFortessa X-20 flow cytometer (BD Biosciences, San Jose, U.S.A.) using the appropriate lasers and band-pass filters (Table 2) and FACSDiva software version 8 (BD Biosciences). Data were analysed using FlowJo software version 10 (BD Biosciences) with gating based on fluorescence minus one controls and populations defined as shown (Table 3).

**Table 2:** Reagents used for immunophenotyping

Target	Clone	Fluorochrome	Excitation wavelength (nm)	Band pass filter wavelength (nm)
hCD127	HIL-7R-M21	BV421	405	450/50
hCD3	UCHT1	BV510	405	525/50
hCD161	HP-3G10	BV605	405	610/20
hCD279 (PD-1)	EH12.1	BV605	405	610/20
hCD183	1C6/CXCR3	BV711	405	710/50
hCD45	HI30	FITC	488	525/15
hCD158e1 (KIR)	DX9	FITC	488	525/15
mCD45	30-F11	PerCP	488	695/40
hCD4	SK3	PerCP-Cy 5.5	488	695/40
hCD25	M-A251	PE	561	586/15
hCD8	RPA-T8	PE-Cy7	561	780/60
hCD56	B159	PE-Cy7	561	780/60
hCD39	TU66	APC	640	670/30
hCD223 (LAG3)	7H2C65	APC	640	670/30
Live/dead stain		Zombie NIR dye	640	780/60

All fluorochrome-conjugated monoclonal antibodies were obtained from BD Biosciences except for CD158e1 and CD223, which were from BioLegend.

APC, allophycocyanin. BV, Brilliant Violet. FITC, fluorescein isothiocyanate. PE, R-phycoerythrin. PE-Cy7, phycoerythrin-cyanine 7. PerCP, perdinin chlorophyll protein-cyanine. PerCP-Cy 5.5, peridinin chlorophyll protein-cyanine5.5. h, human. m, mouse. NIR, near infrared..

**Table 3:** Markers used to define immune cell populations

Population	Markers used to define population
hCD45^+^	hCD45^+^mCD45^−^
hCD3^+^	hCD45^+^hCD3^+^
hCD4^+^	hCD45^+^hCD3^+^hCD4^+^
hCD8^+^	hCD45^+^hCD3^+^hCD8^+^
hTregs	hCD45^+^hCD3^+^hCD4^+^hCD127^low^hCD25^+^
hCD39^+^hTregs	hCD45^+^hCD3^+^hCD127^low^hCD25^+^hCD39^+^
hTcons	hCD45^+^hCD3^+^hCD4^+^ cells excluding hTregs
hTh17	hCXCR3^-^hCD161^+^ hTcons
hTh1	hCXCR3^+^hCD161^−^ hTcons
hTc17	hCD45^+^hCD3^+^hCD8^+^hCD161^high^
hKIR^+^hCD8^+^	hCD45^+^hCD3^+^hCD8^+^hCD158e1^+^
hUnconv T	hCD45^+^hCD3^+^hCD56^+^
hNK	hCD45^+^hCD3^−^hCD56^+^

h, human. Treg, regulatory T cell. Th, T helper. Tc, cytotoxic T. Uncov, unconventional. NK, natural killer. Tcon, conventional T. KIR, killer cell immunoglobulin-like receptor.

### Data presentation and statistical analysis

Data are presented as the mean ± standard error of the mean. Data from mice that were euthanised prior to experimental endpoint were carried forward. Mice (*n*=7) with <5% human CD45^+^ cells in tissues examined by flow cytometry were considered non-engrafted and excluded from analyses. Data sets were tested for normality with a Shapiro–Wilk test. Statistical differences for multiple comparisons were analysed using one-way analysis of variance (ANOVA) with Dunnett’s post-hoc (parametric) or Kruskal–Wallis with Dunn’s multiple comparisons correction (non-parametric) tests. Statistical differences in clinical score and weight over time were assessed using a repeated measures two-way ANOVA with Geisser–Greenhouse correction. Statistical differences in survival were assessed using a Mantel–Cox test. *P* values ≤ 0.1 are reported on graphs. *P* values reported in the corner of weight, clinical score and survival graphs represent the difference between all treatment groups. Statistical analyses and graphs were produced using GraphPad Prism software version 9 (GraphPad Software, La Jolla, U.S.A.). Differences were considered significant where *P*<0.05.

## Results

### Low-dose PTCy prolongs time to GVHD onset in humanised NSG mice

**Figure 1:**
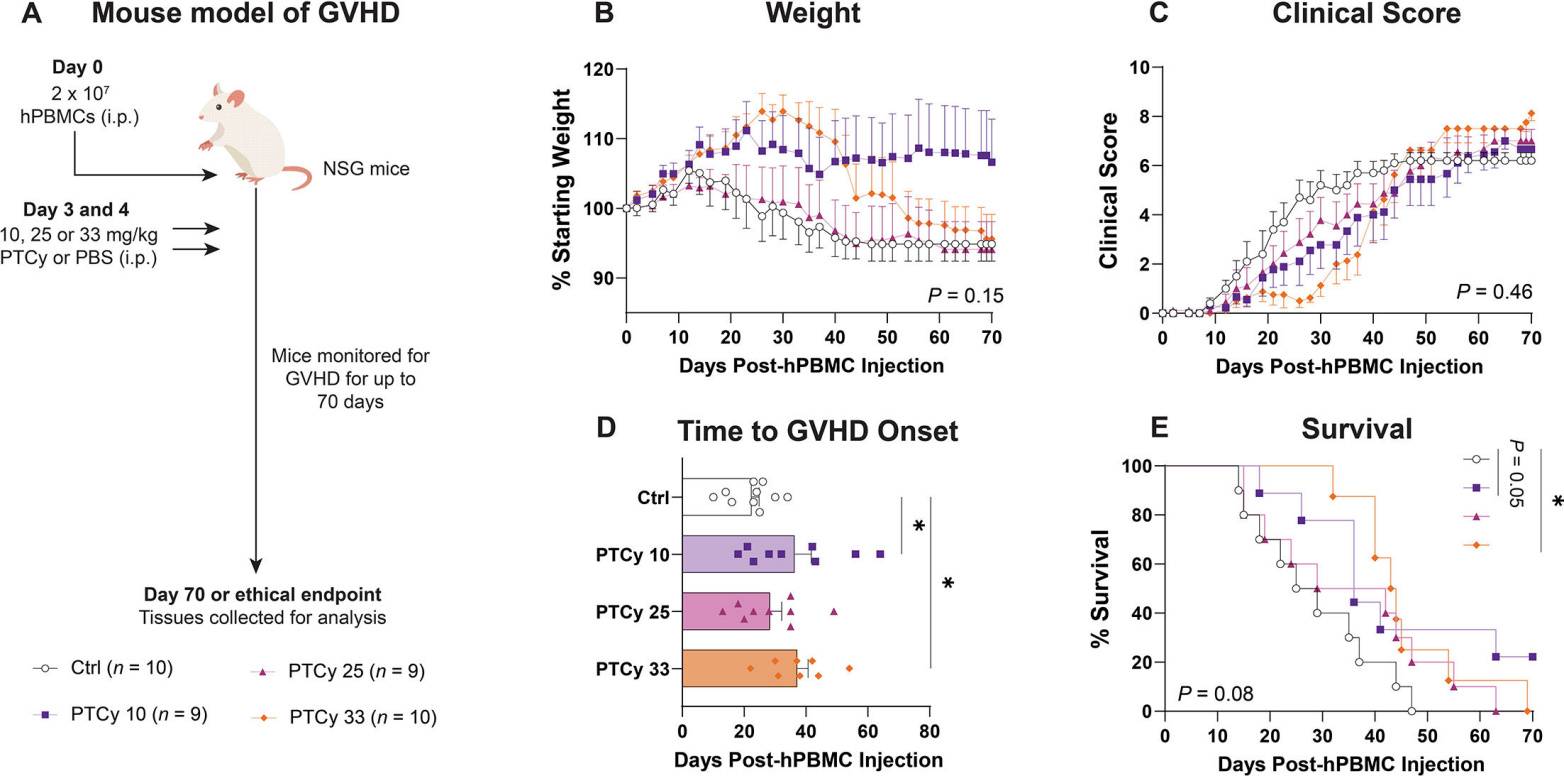
Low-dose PTCy prolongs time to GVHD onset in humanised NSG mice. (**A**) Schematic overview of the humanised mouse model. NSG mice were injected i.p. with 2 × 10^7^ hPBMCs (*n*=6 donors) and subsequently injected i.p. on days 3 and 4 post-hPBMC injection with cyclophosphamide (PTCy) at 10 mg/kg (PTCy 10), 25 mg/kg (PTCy 25) or 33 mg/kg (PTCy 33), or with PBS (Ctrl). Mice were monitored for clinical signs of GVHD, including (**B**) weight and (**C**) clinical score, over 70 days. (**D**) Time to GVHD onset (defined as a clinical score ≥ 3) and (**E**) survival were also assessed. Data are presented as the mean ± standard error of the mean (*n*=8–10). Data are from two independent experiments with two or six donors. Significance was analysed using (**B, C**) two-way ANOVA, (**D**) one-way ANOVA and (**E**) log-rank (Mantel–Cox) tests, with normality tested using a Shapiro–Wilk test. *P<0.05.

Our group has previously shown that high-dose PTCy (33 mg/kg) on days 3 and 4 reduces but does not prevent GVHD in humanised NSG mice, acting through the depletion of proliferating T cells observed at day 6 [44]. However, high-dose PTCy is associated with toxicity risks [15,16,45]. To determine whether lower doses of PTCy could attenuate GVHD, NSG mice were injected i.p. with 2 × 10^7^ hPBMCs (from six healthy donors) on day 0. Mice then received i.p. injections of PTCy at a low dose (10 mg/kg; PTCy 10), mid dose (25 mg/kg; PTCy 25) or high dose (33 mg/kg; PTCy 33) or PBS for control (Ctrl) mice on days 3 and 4. A dose of 33 mg/kg PTCy was considered high-dose due to the established use of this dose in this model, mechanistic relevance and tolerability rather than direct mg/kg equivalence to the clinical 50 mg/kg dose. Mice were subsequently monitored for up to 70 days for clinical signs of GVHD (Figure 1A).

Weight loss was observed in Ctrl mice from day 12, whereas PTCy 33 mice gained weight until day 30 before steadily declining in weight until endpoint (Figure 1B). PTCy 10 mice gained weight until day 20 before plateauing and maintaining weight until endpoint, whereas PTCy 25 mice began to lose weight from day 12, similar to Ctrl mice. However, the overall difference between groups was not statistically significant (*P*=0.15).

Clinical scores of Ctrl, PTCy 10 and PTCy 25 mice increased from day 9, whereas there was a delayed increase in the clinical scores of PTCy 33 mice, beginning at day 30. Nevertheless, the overall difference between groups was not statistically significant (*P*=0.46) and all mice had similar clinical scores at endpoint (*P*=0.35) (Figure 1C).

Time to GVHD onset (defined as a clinical score ≥ 3) [46] was similar between Ctrl (23 days) and PTCy 25 mice (28 days) (*P*=0.55). When compared with Ctrl mice, time to GVHD onset was significantly delayed in PTCy 10 (36 days) (*P*=0.03) and PTCy 33 mice (37 days) (*P*=0.04) (Figure 1D).

Ctrl mice had a median survival time (MST) of 27 days. Comparatively, survival was prolonged in PTCy 10 (MST=36 days) (*P*=0.05), PTCy 25 (MST=35.5 days) (*P*=0.21) and PTCy 33 mice (MST=43.5 days) (*P*=0.03) (Figure 1E). Notably, 30% of PTCy 10 mice survived until the endpoint (day 70).

### PTCy at any dose does not improve histological outcomes at endpoint

**Figure 2:**
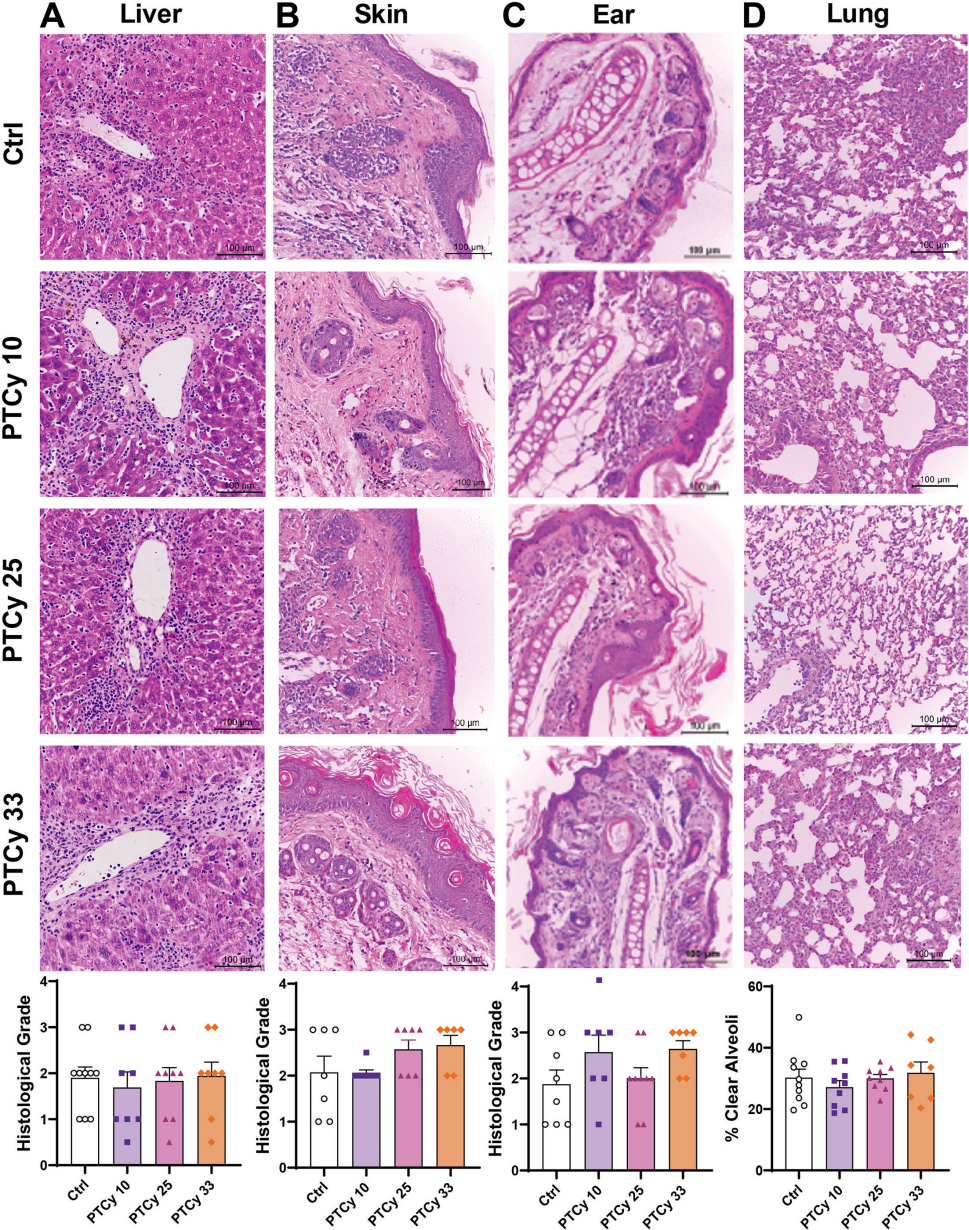
PTCy at any dose does not improve histological outcomes at endpoint. At day 70 or ethical endpoint, organs were sectioned (3–5 µm) and stained with haematoxylin and eosin. Sections of (**A**) liver, (**B**) skin, (**C**) ear and (**D**) lung were examined for histological GVHD. (**A-C**) Liver, skin and ear were assessed using a standardised grading system and (**D**) lung was assessed as the percentage of clear alveoli area of total lung area. Images are representative of 7–10 mice per treatment group. Data are presented as the mean ± standard error of the mean (*n*=7–10). Data are from two independent experiments. Normality was assessed using a Shapiro–Wilk test and significance was analysed using (**A-C**) Kruskal–Wallis and (**D**) one-way ANOVA tests.

We have previously observed reduced GVHD-associated liver histopathology in humanised NSG mice treated with high-dose PTCy (33 mg/kg) [44]. However, the effects of lower doses of PTCy on histological outcomes in this model have not been assessed. Thus, histology of liver, skin, ears and lungs was performed at day 70 or the ethical endpoint to compare the effects of each PTCy dose on late-stage GVHD in humanised NSG mice (Figure 2).

Substantial immune cell infiltration and tissue damage were observed in the liver of Ctrl mice (histological grades 0.5–3, mean>1.5) (Figure 2A). Treatment with either low-, mid- or high-dose PTCy did not reduce GVHD-associated liver histopathology (histological grades 0.5–3, mean>1.5) (*P*=0.93). Moreover, histological evidence of GVHD was similar among all groups in the skin (histological grades 1–3, mean>2) (*P*=0.14) (Figure 2B) and ear (histological grades 1–4, mean>1.5) (*P*=0.16) (Figure 2C), with mice across all groups experiencing epidermal thickening and immune cell infiltration. The mean percentage of clear alveoli space (an inverse measure of lung GVHD) in the lungs of Ctrl mice was 30.2%, and this was similar in mice treated with each dose of PTCy (27.1–31.8%) (*P*=0.64) (Figure 2D).

### Low- and mid-dose PTCy do not affect splenic human leukocyte subsets at endpoint

**Figure 3:**
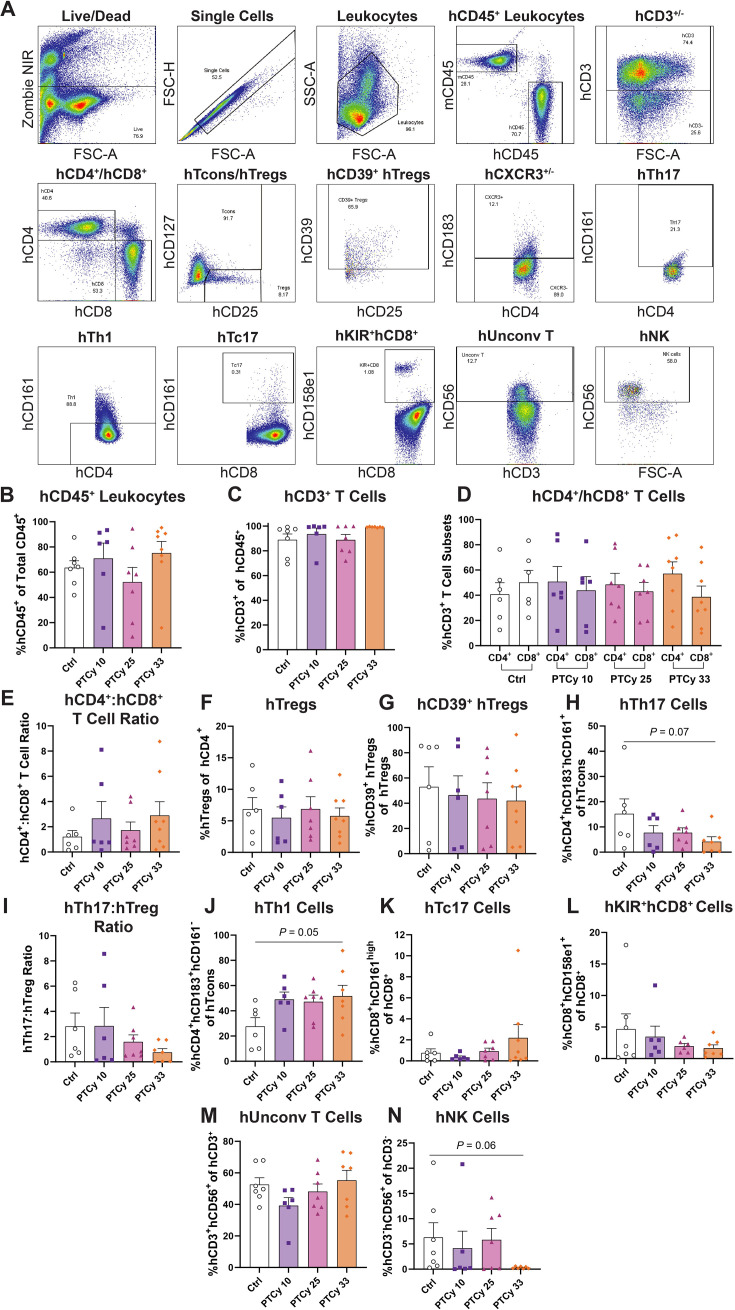
Low- and mid-dose PTCy do not affect splenic human leukocyte subsets at endpoint. (**A**) Spleens from humanised mice were collected at day 70 or ethical endpoint and immune cell subsets were analysed by flow cytometry using a consistent gating strategy. Live cells were gated based on Zombie near infrared (NIR) staining. Single cells were gated based on forward scatter area (FSC-A) and forward scatter height (FSC-H) before viable human (**h**) and mouse (**m**) leukocytes were gated using FSC-A and side scatter area (SSC-A). hCD45^+^ and mCD45^+^ leukocytes were then gated before identifying hCD3^+^ and hCD3^−^ cells. Within the hCD3^+^ population, hCD4^+^ and hCD8^+^ T cells were gated before identifying hCD25^+^hCD127^low^ hTregs, hCD39^+^ hTregs, hCD8^+^hCD161^high^ hTc17 cells, hCD8^+^hCD158e1^+^ hKIR^+^hCD8^+^ cells and hCD3^+^hCD56^+^ hUnconventional (hUnconv) T cells. hCD183^+/−^ (CXCR3) cells were gated before hCD4^+^hCD183^−^hCD161^+^ hTh17 cells hCD4^+^hCD183^+^hCD161^−^ hTh1 cells were identified. Within the hCD3^−^ population, hCD3^−^hCD56^+^ NK cells were identified. (**B**) hCD45^+^ and mCD45^+^ leukocytes were gated before identifying (**C**) hCD3^+^ T cells, (**D**) hCD4^+^ and hCD8^+^ T cell subsets, (**E**) hCD4^+^:hCD8^+^ T cell ratios, (**F**) hTregs, (**G**) hCD39^+^ hTregs, (**H**) hTh17 cells, (**I**) hTh17:hTreg ratios, (**J**) hTh1 cells, (**K**) hTc17 cells, (**L**) hKIR^+^hCD8^+^ cells, (**M**) hUnconv T cells and (**N**) hNK cells. Data are presented as the mean ± standard error of the mean (*n*=6–8). Symbols represent individual mice. Data are from two independent experiments. Normality was assessed using a Shapiro–Wilk test and significance was tested using (**D, F, H, I, J, M**) one-way ANOVA or (**B, C, E, G, K, L, N**) Kruskal–Wallis tests.

High-dose PTCy (33 mg/kg) can alter the proportions of human (h) leukocytes in humanised NSG mice [44,47]; however, the impact of low- and mid-dose PTCy on human leukocyte subsets in this model is yet to be explored. Therefore, human immune cell subsets in spleens from the humanised mice above were assessed by flow cytometry (using a consistent gating strategy) (Figure 3A) at day 70 or the ethical endpoint.

PTCy at any dose did not affect engraftment of hCD45^+^ leukocytes, with each group showing similar proportions (52.2–75.2%) compared with Ctrl mice (63.6%) (*P*=0.21) (Figure 3B). These cells were predominantly hCD3^+^ T cells with similar proportions in all four groups (88.8–99.2%) (*P*=0.20) (Figure 3C). The splenic hCD4^+^:hCD8^+^ T cell ratio correlates with clinical GVHD severity in both humans [48,49] and in humanised NSG mice [46]. Proportions of hCD4^+^ and hCD8^+^ T cells were similar in all four groups (*P*=0.86) (Figure 3D), resulting in similar hCD4^+^:hCD8^+^ T cell ratios (1.2–2.9) (*P*=0.72) (Figure 3E).

Tregs can reduce GVHD severity by inhibiting the activation and proliferation of effector T cells [35,50]. PTCy at any dose did not affect the proportions of splenic hTregs (hCD4^+^hCD25^+^hCD127^low^) (5.5–6.9%) compared with Ctrl mice (6.9%) (*P*=0.91) (Figure 3F). Proportions of highly suppressive hCD39^+^ hTregs [51] were also similar across all groups (42.1–46.5%) (*P*=0.94) (Figure 3G). Notably, mice reconstituted with hPBMCs from different donors exhibited variable proportions of hCD39^+^hTregs, with some donors resulting in markedly lower proportions in recipient mice.

Th17 cells play a pathogenic role in GVHD [52]. Compared with Ctrl mice (15.3%), proportions of splenic hTh17 cells (hCD4^+^hCD183^−^hCD161^+^) were similar in PTCy 10 and PTCy 25 mice (7.7%) (*P*=0.30 and *P*=0.28, respectively). However, splenic hTh17 cells were reduced by 72% in PTCy 33 mice, which approached statistical significance (*P*=0.07) (Figure 3H). Compared with Ctrl mice, the hTh17:hTreg ratio was similar in PTCy-treated mice (*P*=0.30) (Figure 3I).

Th1 cell maturation is a dominant feature of GVHD development, with elevated Th1 cytokines correlating with greater disease severity and earlier onset in preclinical models [53,54]. Compared with Ctrl mice (27.7%), proportions of hTh1 cells (hCD4^+^hCD183^+^hCD161^−^) were approximately two-fold higher in PTCy 10, PTCy 25 (*P*=0.11 and *P*=0.13, respectively) and PTCy 33 mice (59.1–51.7%), which approached statistical significance in the latter group only (*P*=0.05) (Figure 3J).

Cytotoxic T (Tc) 17 cells (hCD8^+^hCD161^high^) can induce GVHD [55]. Apart from one mouse in the PTCy 33 group, proportions of hTc17 cells were relatively low and similar across all groups (0.76–2.19%) (*P*=0.73) (Figure 3K).

Human killer cell immunoglobulin-like receptor (KIR)^+^CD8^+^ T cells, equivalent to mouse Ly49^+^ CD8^+^ Tregs, are a recently identified subset that are particularly active during infections and are thought to regulate self-reactive T cells that are activated by infection [56,57]. However, the role of these regulatory cells in GVHD is unknown. Excluding one mouse in each of the Ctrl and PTCy 10 groups, proportions of splenic hKIR^+^hCD8^+^ cells (hCD8^+^hCD158e1^+^) were similar across all groups (1.5–4.7%) (*P*=0.75) (Figure 3L).

CD3^+^CD56^+^ T cells are a heterogeneous population of unconventional (hUnconv) T cells, including mucosal-associated invariant T cells, γδ T cells and natural killer (NK) T cells [58]. In the context of GVHD, the role of hUnconv T cells is not fully elucidated, but their dual functionality suggests they could contribute to both the pathogenesis and regulation of GVHD [59–61]. PTCy, at any dose, did not affect proportions of hUnconv T cells (hCD3^+^hCD56^+^), with similar proportions observed in all groups (39.2–55.3%) (*P*=0.19) (Figure 3M).

NK cells are the primary human non-T cell population to engraft in this model [62] and can suppress GVHD in mice while promoting GVL immunity [63]. Compared with Ctrl mice, proportions of hNK cells (hCD3^−^hCD56^+^) were similar in PTCy 10 and PTCy 25 mice (4.2–6.3%) (*P*=0.32 and *P*>0.99, respectively) but were reduced by 95.2% in PTCy 33 mice, which approached statistical significance (*P*=0.06) (Figure 3N).

### PTCy at any dose does not affect hepatic human leukocyte subsets at endpoint

The liver is a primary target organ of GVHD in both humans [64] and humanised NSG mice [65]. Therefore, human immune cell subsets in livers from the humanised mice above were assessed by flow cytometry (using a consistent gating strategy) (Figure 3A) at day 70 or the ethical endpoint.

**Figure 4:**
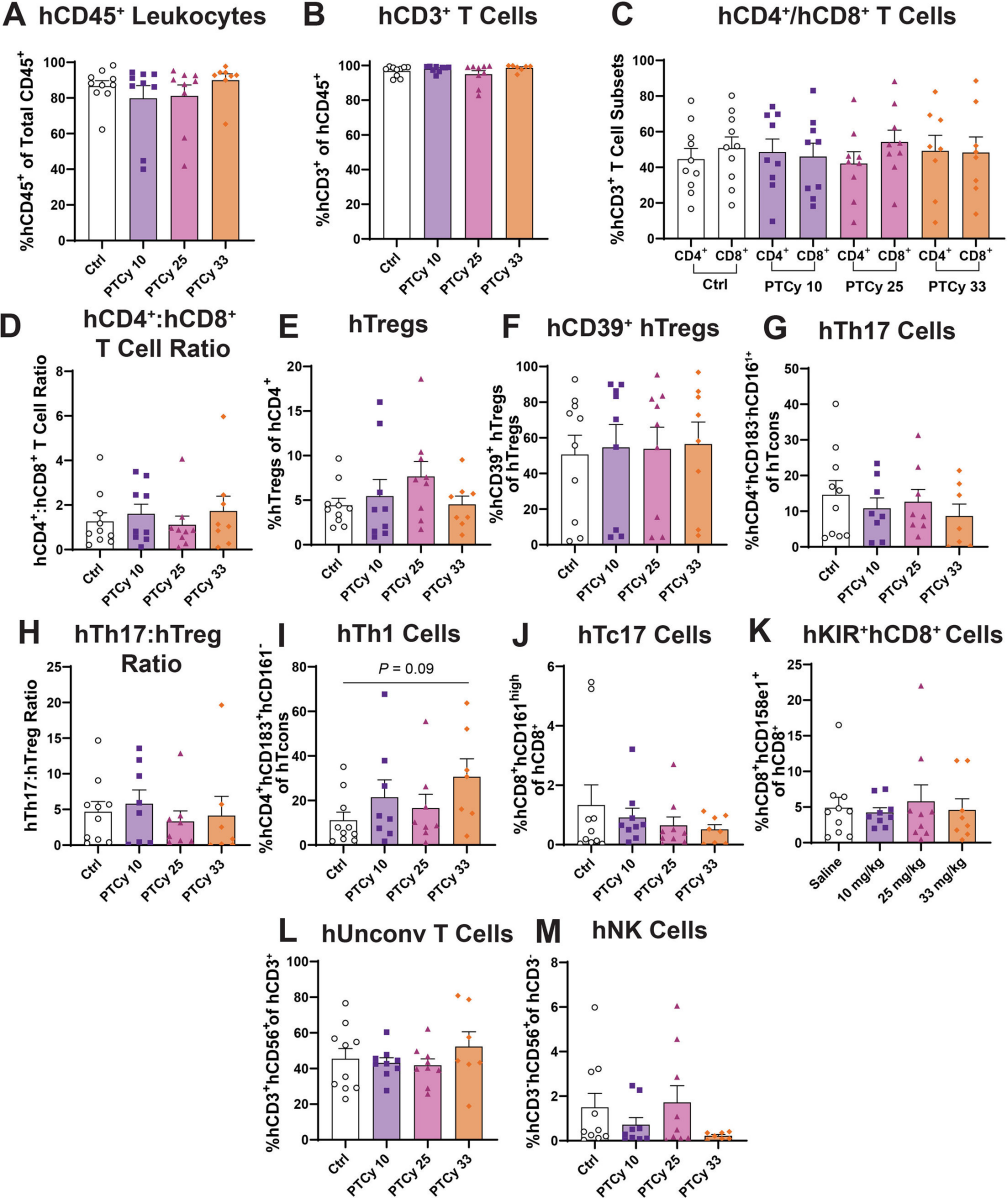
PTCy at any dose does not affect hepatic human leukocyte subsets at endpoint. Livers from humanised mice were collected at day 70 or the ethical endpoint, and immune cell subsets were analysed by flow cytometry using the gating strategy shown in Figure 3A (**A**) hCD45^+^ and mCD45^+^ leukocytes were gated before identifying (**B**) hCD3^+^ T cells, (**C**) hCD4^+^ and hCD8^+^ T cell subsets, (**D**) hCD4^+^:hCD8^+^ T cell ratios, (**E**) hTregs, (**F**) hCD39^+^ hTregs, (**G**) hTh17 cells, (**H**) hTh17:hTreg ratios, (**I**) hTh1 cells, (**J**) hTc17 cells, (**K**) hKIR^+^hCD8^+^ cells, (**L**) hUnconv T cells and (**M**) hNK cells. Data are presented as the mean ± standard error of the mean (*n*=7–10). Symbols represent individual mice. Data are from two independent experiments. Normality was assessed using a Shapiro–Wilk test and significance was tested using (**C, L**) one-way ANOVA or (**A, B, D, E, F, G, H, I, J, K, M**) Kruskal–Wallis tests.

As observed in the spleen (Figure 3), PTCy at any dose did not affect proportions of hCD45^+^ leukocytes in the liver, with all groups showing similar proportions to Ctrl mice (79.8–90.0%) (*P*=0.36) (Figure 4A). These cells were predominantly hCD3^+^ T cells, with similar proportions in all four groups (94.9–98.6%) (*P*=0.20) (Figure 4B). Furthermore, proportions of hCD4^+^ and hCD8^+^ T cells were similar in all four groups (*P*=0.96) (Figure 4C), as were the hCD4^+^:hCD8^+^ T cell ratios (1.1–1.7) (*P*=0.92) (Figure 4D).

In the liver, PTCy at any dose did not affect proportions of hTregs with similar proportions in PTCy-treated (4.5–7.7%) and Ctrl mice (4.4%) (*P*=0.33) (Figure 4E). Furthermore, proportions of hCD39^+^ Tregs were also similar across all four groups (50.6–56.4%) (*P*=0.89), again with notable variation related to donors used (Figure 4F).

Proportions of hepatic hTh17 cells were similar between Ctrl (14.6%) and PTCy-treated mice (8.6–10.8%) (*P*=0.68) (Figure 4G), as were hTh17:hTreg ratios (3.3–5.8) (*P*=0.65) (Figure 4H). Compared with Ctrl mice (11.1%), proportions of hepatic hTh1 cells were similar in PTCy 10 (21.4%) and PTCy 25 (16.6%) mice (*P*=0.75 and *P*>0.99, respectively) but were increased approximately three-fold in PTCy 33 (30.6%) mice, which approached statistical significance (*P*=0.09) (Figure 4I).

Apart from two mice in the Ctrl group, proportions of hTc17 cells were low (0.5–1.3%) and similar in all four groups (*P*=0.62) (Figure 4J). Compared with Ctrl mice (4.9%), proportions of hKIR^+^hCD8^+^ cells were similar in PTCy-treated groups (4.2–5.8%) (*P*=0.94) (Figure 4K).

Compared with Ctrl mice (45.4%), proportions of hUnconv T cells were similar in PTCy-treated groups (41.8–52.3%) (*P*=0.56) (Figure 4L). Proportions of hNK cells were similar between Ctrl (1.5%) and PTCy-treated mice (0.2–1.7%) (*P*=0.56) (Figure 4M).

### PTCy doses differentially affect splenic human PD-1^+^ T cell subsets at endpoint

**Figure 5:**
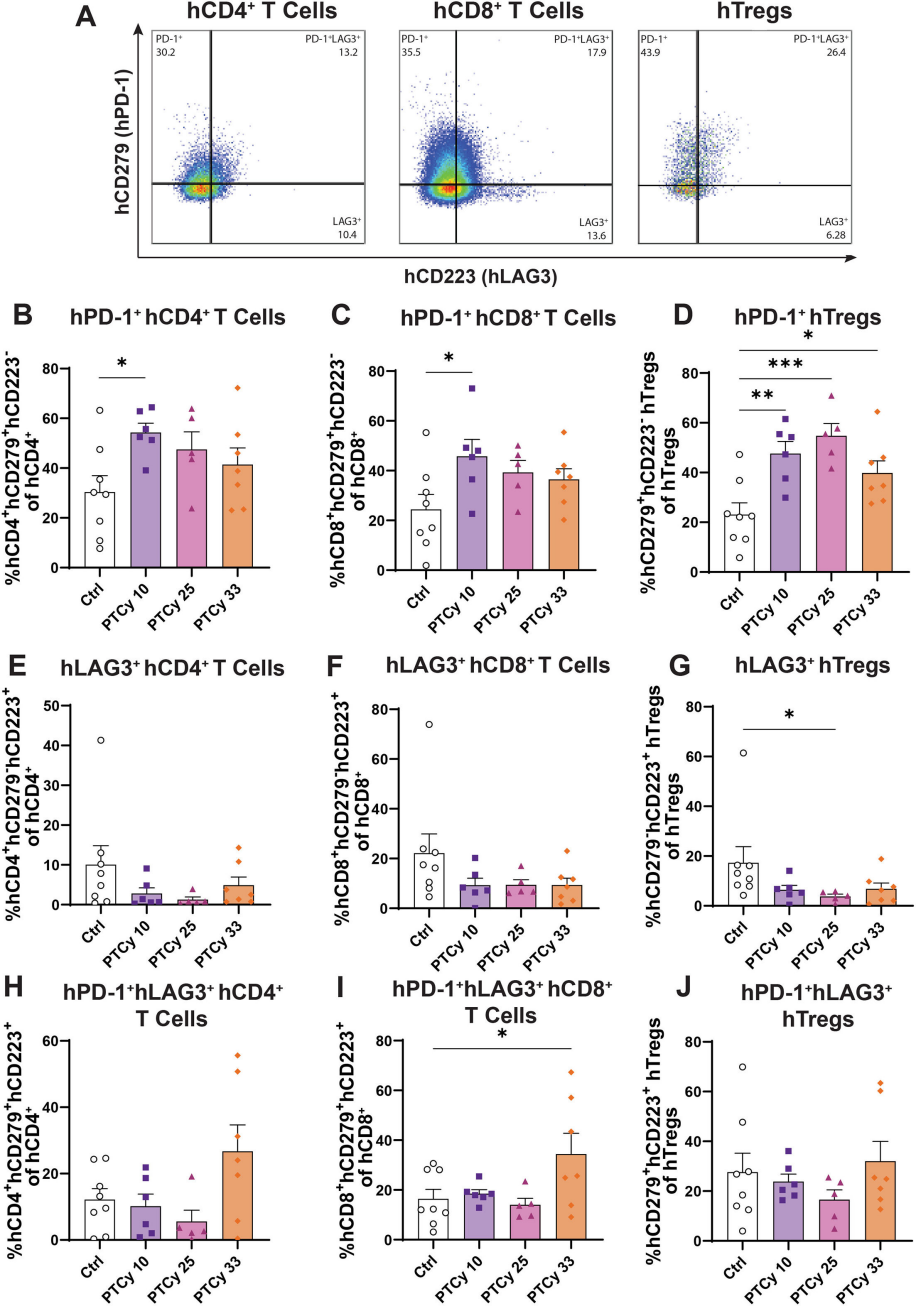
PTCy doses differentially affect splenic human PD-1^+^ T cell subsets at endpoint. Spleens from humanised mice were collected at day 70 or ethical endpoint and were examined by flow cytometry. Proportions of human (**H**) CD45^+^ leukocytes, hCD3^+^ T cells, hCD4^+^ and hCD8^+^ T cell subsets and hTregs were identified using the gating strategy shown in Figure 3A before assessing (**A**) hCD279^+^hCD223^-^ (hPD-1^+^), hCD279^-^hCD223^+^ (hLAG3^+^) and hCD279^+^hCD223^+^ (hPD-1^+^hLAG3^+^) subsets as shown. Proportions of (**B**) hPD-1^+^hCD4^+^ T cells, (**C**) hPD-1^+^hCD8^+^ T cells, (**D**) hPD-1^+^hTregs, (**E**) hLAG3^+^hCD4^+^ T cells, (**F**) hLAG3^+^hCD8^+^ T cells, (**G**) hLAG3^+^hTregs, (**H**) hPD-1^+^hLAG3^+^hCD4^+^ T cells, (**I**) hPD-1^+^hLAG3^+^hCD8^+^ T cells and (**J**) hPD-1^+^hLAG3^+^hTregs were assessed. Data are presented as the mean ± standard error of the mean (*n*=5–8). Data are from two independent experiments. Normality was assessed using a Shapiro–Wilk test and significance was tested using (**B, C, D, I**) one-way ANOVA or (**E, F, G, H, J**) Kruskal–Wallis tests. ***P<0.001, **P<0.01, *P<0.05.

The precise mechanisms through which PTCy reduces GVHD remain incompletely understood. Previous studies have indicated that PTCy may work via the induction of effector T cell dysfunction or exhaustion [19,66], but there is limited data investigating this notion. As such, the effects of low-, mid- and high-dose PTCy on splenic T cell subsets expressing co-inhibitory receptors PD-1 and LAG3, which are known markers for exhausted T cells [27,29], were assessed by flow cytometry at day 70 or the ethical endpoint. These markers were assessed using the above gating strategy (as shown in Figure 3A) before identifying hPD-1^+^ and hLAG3^+^ cells using a consistent gating strategy (Figure 5A).

Compared with Ctrl mice (30.4%), proportions of splenic hPD-1^+^hCD4^+^ T cells were significantly increased 1.7-fold in low-dose PTCy 10 mice (*P*=0.03) but were similar in PTCy 25 (*P*=0.21) and PTCy 33 mice (*P*=0.48) (Figure 5B). Compared with Ctrl mice (24.5%), proportions of splenic hPD-1^+^hCD8^+^ T cells were also significantly increased 1.9-fold in PTCy 10 mice (*P*=0.03) but were similar in PTCy 25 (*P*=0.21) and PTCy 33 mice (*P*=0.29) (Figure 5C). In contrast, compared with Ctrl mice (23.1%), proportions of splenic hPD-1^+^hTregs were significantly increased by 2.1-fold in PTCy 10 (*P*=0.004), 2.4-fold in PTCy 25 (*P*=0.0006) and 1.7-fold in PTCy 33 mice (*P*=0.04) (Figure 5D).

Compared with Ctrl mice (10.1%), proportions of splenic hLAG3^+^hCD4^+^ T cells were similar in PTCy-treated mice (*P*=0.18) (Figure 5E). Similarly, compared with Ctrl mice (22.1%), proportions of splenic hLAG3^+^hCD8^+^ T cells were similar in PTCy-treated mice (*P*=0.29) (Figure 5F). Proportions of hLAG3^+^hTregs were similar in PTCy 10 (*P*=0.12) and PTCy 33 mice (*P*=0.18) but were significantly reduced by 78.1% in PTCy 25 mice (*P*=0.02) compared with Ctrl mice (17.3%) (Figure 5G).

Proportions of splenic hPD-1^+^hLAG3^+^hCD4^+^ T cells were similar in Ctrl (12.2%) and PTCy-treated mice (1.3–4.9%) (*P*=0.15) (Figure 5H). Markedly, compared with Ctrl mice (16.4%), proportions of splenic hPD-1^+^hLAG3^+^hCD8^+^ T cells were similar in PTCy 10 (18.5%) and PTCy 25 mice (14.1%) (*P*=0.99 and *P*=0.98, respectively), but were significantly increased 2.1-fold in PTCy 33 mice (34.5%) (*P*=0.04) (Figure 5I). Proportions of splenic hPD-1^+^hLAG3^+^hTregs were similar across all four groups (16.5–32.1%) (*P*=0.65) (Figure 5J).

### PTCy doses do not differentially affect hepatic human PD-1^+^ T cell subsets at endpoint

**Figure 6:**
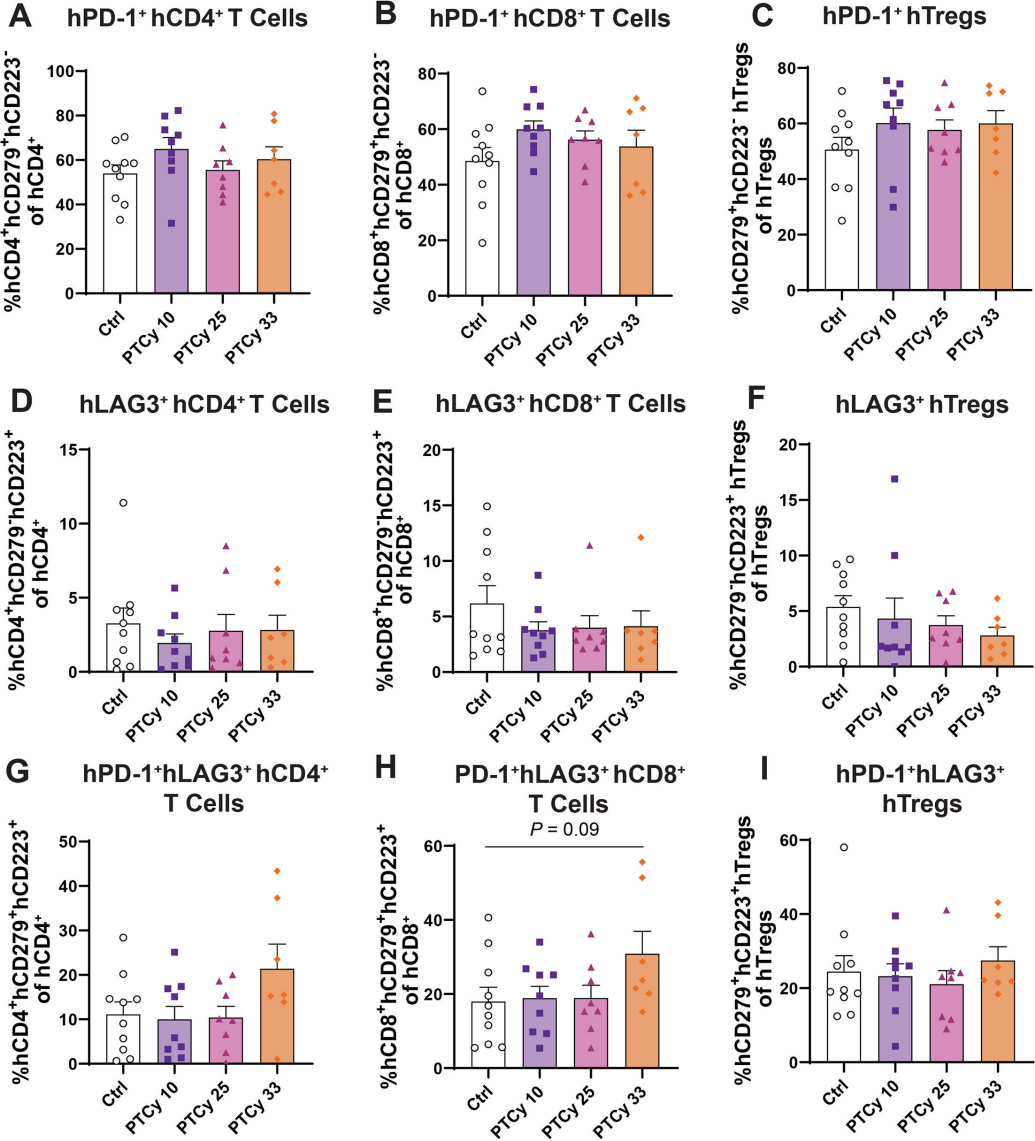
PTCy doses do not differentially affect hepatic human PD-1^+^ T cell subsets at endpoint. Livers from humanised mice were collected at day 70 or the ethical endpoint and were examined by flow cytometry. Proportions of human (h) CD45^+^ leukocytes, hCD3^+^ T cells, hCD4^+^ and hCD8^+^ T cell subsets and hTregs were identified using the gating strategy shown in Figure 3A before assessing hCD279^+^hCD223^−^ (hPD-1^+^), hCD279^−^hCD223^+^ (hLAG3^+^) and hCD279^+^hCD223^+^ (hPD-1^+^hLAG3^+^) subsets as shown in Figure 5A. Proportions of (**A**) hPD-1^+^hCD4^+^ T cells, (**B**) hPD-1^+^hCD8^+^ T cells, (**C**) hPD-1^+^hTregs, (**D**) hLAG3^+^hCD4^+^ T cells, (**E**) hLAG3^+^hCD8^+^ T cells, (**F**) hLAG3^+^hTregs, (**G**) hPD-1^+^hLAG3^+^hCD4^+^ T cells, (**H**) hPD-1^+^hLAG3^+^hCD8^+^ T cells and (**I**) hPD-1^+^hLAG3^+^hTregs were assessed. Data are presented as the mean ± standard error of the mean (*n*=7–10). Data are from two independent experiments. Normality was assessed using a Shapiro–Wilk test and significance was tested using (**A, B, G, H**) one-way ANOVA or (**C, D, E, F, I**) Kruskal–Wallis tests.

In the liver, proportions of hPD-1^+^hCD4^+^ T cells were similar between Ctrl and low-, mid- and high-dose PTCy-treated mice (53.9–65.0%) (*P*=0.31) (Figure 6A), as were proportions of hPD-1^+^hCD8^+^ T cells (48.6–59.9%) (*P*=0.28) (Figure 6B). Furthermore, proportions of hPD-1^+^hTregs were similar between all four groups (50.6–60.2%) (*P*=0.38) (Figure 6C). Moreover, proportions of hepatic hLAG3^+^hCD4^+^ T cells were relatively low and similar between Ctrl and PTCy-treated mice (1.9–3.3%) (*P*=0.83) (Figure 6D), as were proportions of hLAG3^+^hCD8^+^ T cells (3.8–6.2%) (*P*=0.98) (Figure 6E) and proportions of hLAG3^+^hTregs (2.8–5.4%) (*P*=0.28) (Figure 6F).

In the liver, proportions of hPD-1^+^hLAG3^+^hCD4^+^ T cells were similar in Ctrl (11.1%) and PTCy-treated mice (10.0–22.4%) (*P*=0.10) (Figure 6G). Like the spleen, hepatic proportions of hPD-1^+^hLAG3^+^hCD8^+^  T cells were similar in Ctrl, PTCy 10 and PTCy 25 mice (18.0–18.9%) (*P*=0.99 and *P*=0.99, respectively), but were increased 1.7-fold in PTCy 33 mice (30.9%), which approached statistical significance (*P*=0.09) (Figure 6H). Proportions of hPD-1^+^hLAG3^+^hTregs were similar between Ctrl and low-, mid- and high-dose PTCy-treated mice (21.1–27.5%) (*P*=0.73) (Figure 6I).

### Low-dose PTCy does not improve clinical signs of GVHD early in disease

**Figure 7:**
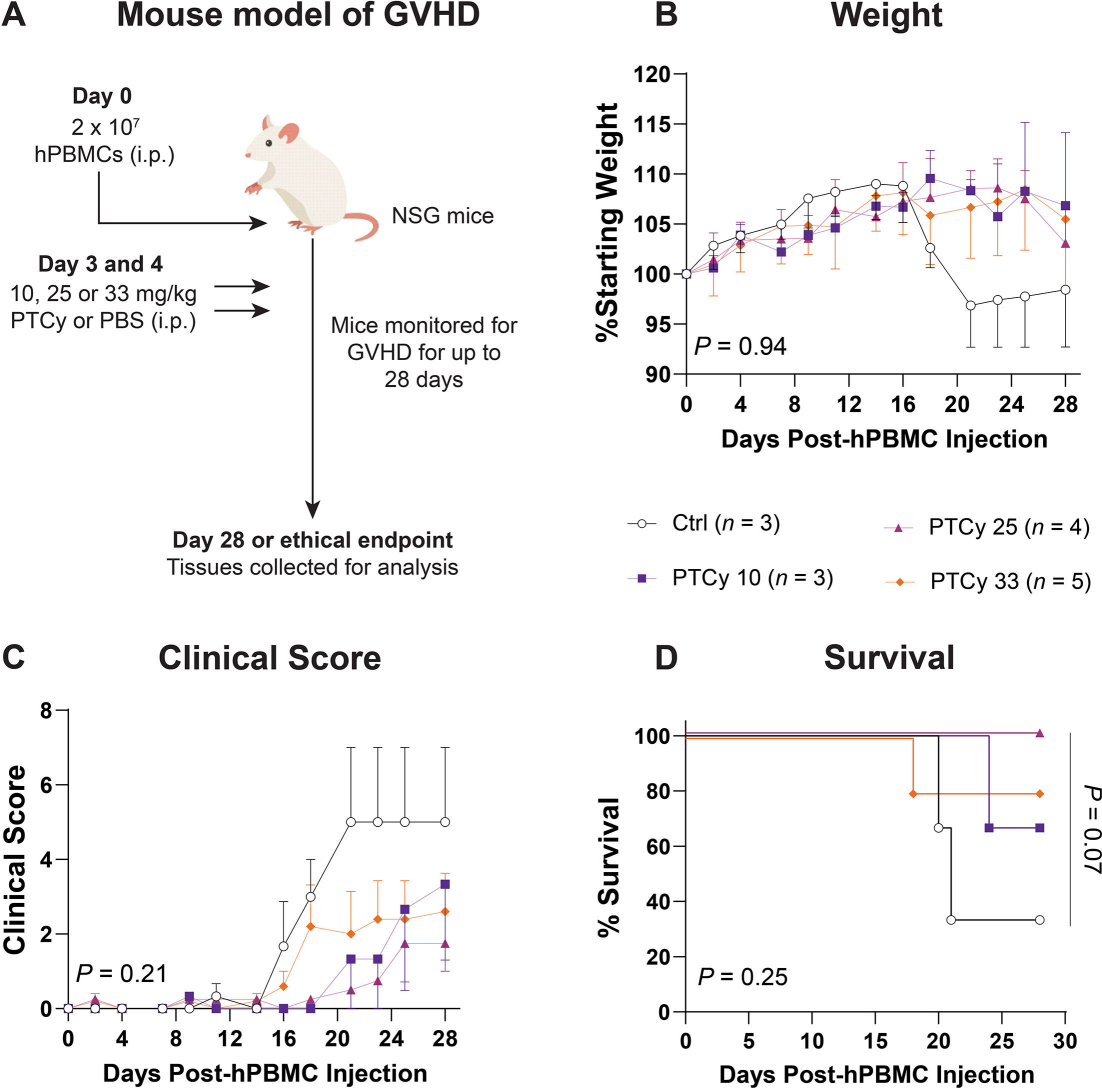
PTCy at any dose does not improve clinical signs of GVHD early in disease. (**A**) Schematic overview of the humanised mouse model. NSG mice were injected i.p. with 2 × 10^7^ hPBMCs (*n*=4 donors) and subsequently injected i.p. on days 3 and 4 post-hPBMC injection with cyclophosphamide (PTCy) at 10 mg/kg (PTCy 10), 25 mg/kg (PTCy 25) or 33 mg/kg (PTCy 33) or with PBS (Ctrl). Mice were monitored for clinical signs of GVHD, including (**B**) weight, (**C**) clinical score and (**D**) survival over 28 days. Data are presented as the mean ± standard error of the mean (*n*=3–5). Data are from one experiment. Significance was analysed using (**B, C**) two-way ANOVA and (**D**) log-rank (Mantel–Cox) tests.

To better understand the mechanisms by which PTCy attenuates GVHD, NSG mice were injected i.p. with hPBMCs (from four healthy donors) and with low, mid or high doses of PTCy or PBS as above (Figure 1) but were euthanised at day 28 during the early stages of GVHD development (Figure 7A).

Weight loss was observed in Ctrl mice from day 18, whereas low-, mid- and high-dose PTCy-treated mice gained and maintained weight until day 28 (Figure 7B). However, the overall difference in weight between groups was not statistically significant (*P*=0.94).

Clinical scores of Ctrl mice increased from day 16 before plateauing from day 20 (Figure 7C). Clinical scores of PTCy 33 mice also increased from day 16, but to a lesser extent than Ctrl mice, plateauing from day 18. Clinical scores of PTCy 10 and PTCy 25 mice increased from day 21. However, there was no statistically significant difference between groups (*P*=0.21).

Compared with Ctrl mice (MST=21 days), survival was prolonged in PTCy 10 (MST>28 days) (*P*=0.30), PTCy 25 (MST>28 days) (*P*=0.07) and PTCy 33 mice (MST>28 days) (*P*=0.30) (Figure 7D).

### Low- and high-dose PTCy partially improve histological liver GVHD early in disease

**Figure 8:**
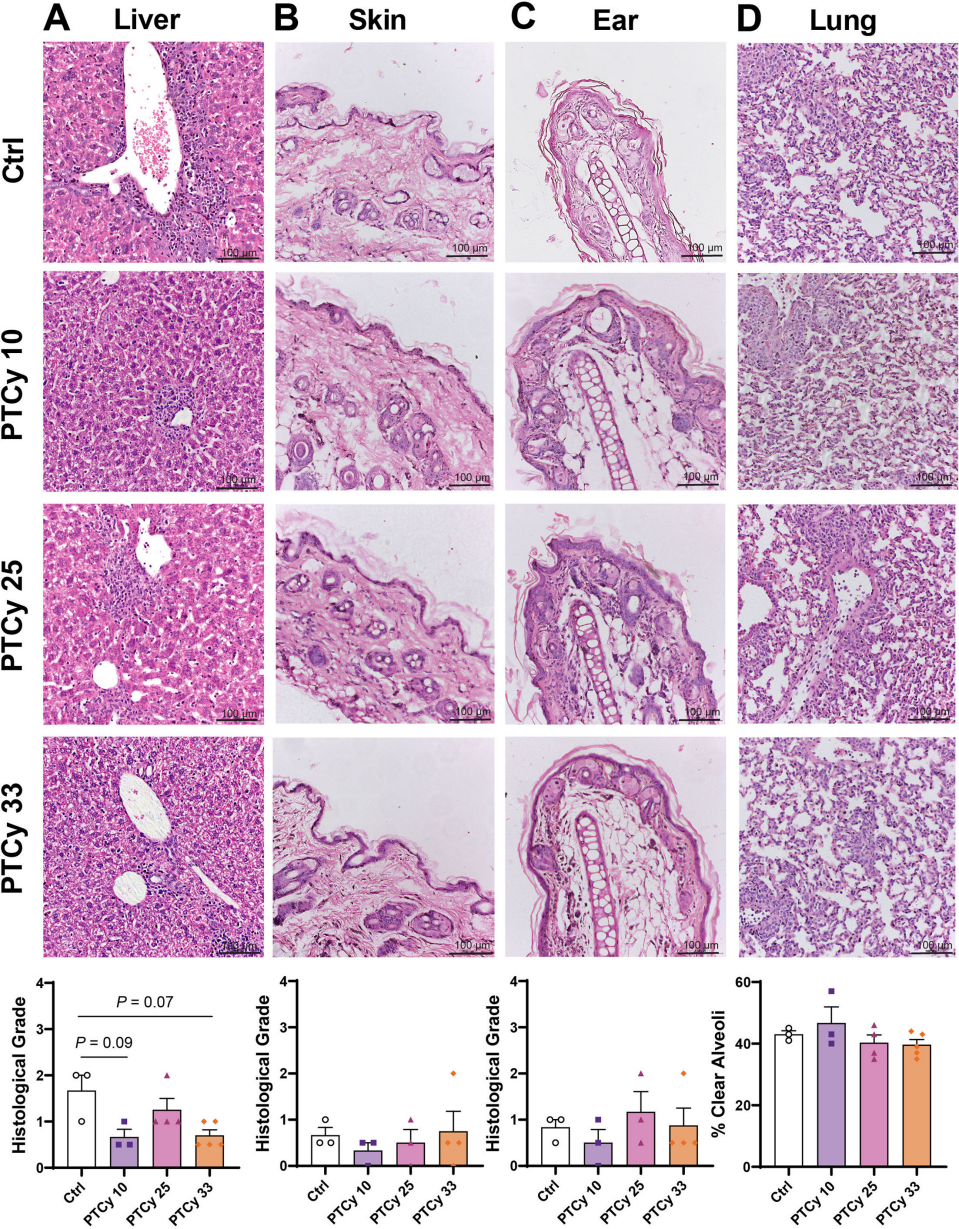
Low- and high-dose PTCy partially improve histological liver GVHD early in disease. At day 28 or the ethical endpoint, organs were sectioned (3–5 µm) and stained with haematoxylin and eosin. Sections of (**A**) liver, (**B**) skin, (**C**) ear and (**D**) lung were examined for histological GVHD. (**A-C**) Liver, skin and ear were assessed using a standardised grading system and (**D**) lung was assessed as the percentage of clear alveoli area of total lung area. Images are representative of 3–5 mice per treatment group. Data are presented as the mean ± standard error of the mean (*n*=3–5). Data are from one experiment. Normality was assessed using a Shapiro–Wilk test and significance was analysed using (**B, D**) one-way ANOVA or (**A, C**) Kruskal–Wallis tests.

Reduced liver histopathology has previously been observed following treatment with high-dose PTCy (33 mg/kg) at day 28 [47], but the effects of low- and mid-dose PTCy on histological GVHD in this model are yet to be explored. Therefore, histology of liver, skin, ears and lungs was performed at day 28 to examine the effects of low- and mid-dose PTCy on early GVHD in humanised NSG mice (Figure 8).

Moderate immune cell infiltration was observed in the liver of Ctrl mice (histological grades 1–2, mean=1.7) (Figure 8A). Compared with Ctrl mice, liver histopathology was reduced in PTCy 10 and PTCy 33 mice (histological grades 0.5–1, mean<1) with values approaching statistical significance (*P*=0.09 and *P*=0.07, respectively), but was similar in PTCy 25 mice (histological grades 1–2, mean=1.3) (*P*>0.99). Histological evidence of GVHD in the skin (histological grades 1–2, mean<1) (Figure 8B) and ear (histological grades 0–2, mean<2) (Figure 8C) was low and similar between Ctrl and PTCy-treated mice (*P*=0.80 and *P*=0.65, respectively), with negligible evidence of epidermal thickening and low to moderate immune cell infiltration across all groups. The mean percentages of clear alveoli space in the lungs of Ctrl mice (43.0%) and in mice treated with low-, mid- or high-PTCy doses (39.6–46.7%) were similar (*P*=0.33) (Figure 8D).

### Mid-dose PTCy increases proportions of splenic human T cells early in disease

To better understand the cellular mechanisms of action by which PTCy attenuates GVHD, the effects of low-, mid- and high-dose PTCy on human leukocyte engraftment in spleens from humanised mice was assessed by flow cytometry, (as shown in Figure 3A) at day 28 or at ethical endpoint.

**Figure 9:**
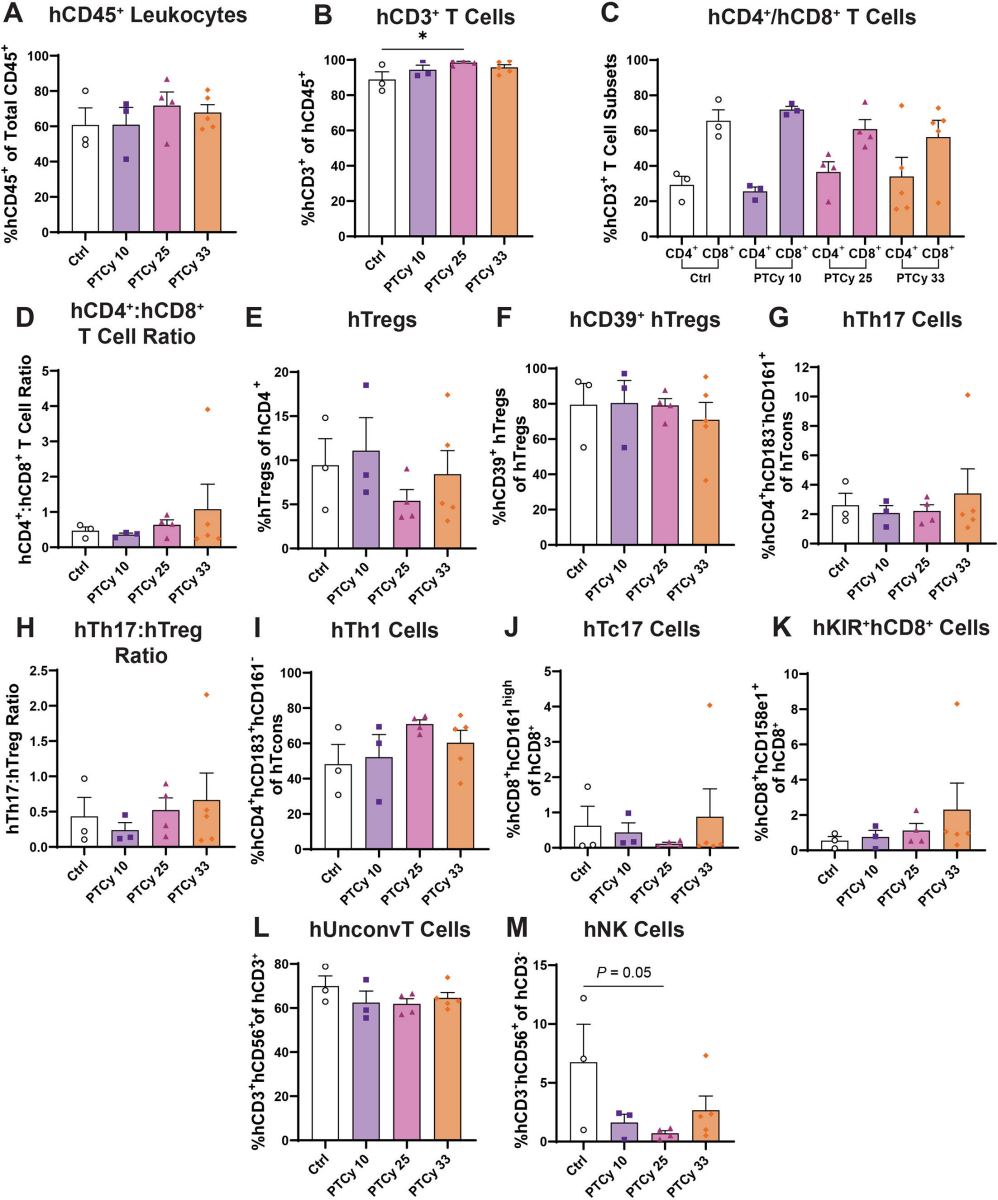
Mid-dose PTCy increases proportions of splenic human T cells early in disease. Spleens from humanised mice were examined by flow cytometry using the gating strategy shown in Figure 3A to identify human (**H**) leukocyte subsets at day 28 or the ethical endpoint. (**A**) hCD45^+^ and mCD45^+^ leukocytes were gated before identifying (**B**) hCD3^+^ T cells, (**C**) hCD4^+^ and hCD8^+^ T cell subsets, (**D**) hCD4^+^:hCD8^+^ T cell ratios, (**E**) hTregs, (**F**) hCD39^+^ hTregs, (**G**) hTh17 cells, (**H**) hTh17:hTreg ratios, (**I**) hTh1 cells, (**J**) hTc17 cells, (**K**) hKIR^+^hCD8^+^ cells, (**L**) hUunconv T cells and (**M**) hNK cells. Data are presented as the mean ± standard error of the mean (*n*=3–5). Symbols represent individual mice. Data are from one experiment. Normality was assessed using a Shapiro–Wilk test and significance was tested using (**A, B, E, F, I, L, M**) one-way ANOVA or (**C, D, G, H, J, K**) Kruskal–Wallis tests. *P<0.05.

In the spleen, PTCy at low, mid or high doses did not affect engraftment of hCD45^+^ leukocytes, with each group showing similar proportions compared with Ctrl mice (60.7–71.7%) (*P*=0.67) (Figure 9A). Proportions of hCD3^+^ T cells were similar between Ctrl (88.8%), PTCy 10 (94.4%) (*P*=0.32) and PTCy 33 mice (95.7%) (*P*=0.13) but were significantly increased by 1.2-fold in PTCy 25 mice (98.5%) compared with Ctrl mice (*P*=0.03) (Figure 9B).

In both Ctrl and low-, mid- or high-dose PTCy-treated mice, splenic proportions of hCD4^+^ T cells (25.5–36.5%) were lower than hCD8^+^ T cells (56.3–71.9%) which were similar across all groups (*P*>0.99) (Figure 9C), as were the splenic hCD4^+^:hCD8^+^ T cell ratios (0.36–1.1) (*P*=0.59) (Figure 9D). PTCy at any dose did not affect splenic proportions of hTregs with similar proportions in PTCy-treated (5.4–11.1%) and Ctrl mice (9.4%) (*P*=0.55) (Figure 9E). Furthermore, proportions of hCD39^+^ Tregs were similar across all four groups (70.8–80.4%) (*P*=0.87) (Figure 9F).

At day 28, proportions of splenic hTh17 cells were relatively low and similar between Ctrl (2.6%) and PTCy-treated mice (2.1–3.4%) (*P*=0.99) (Figure 9G) as were hTh17:hTreg ratios (0.24–0.66) (*P*=0.82) (Figure 9H). Moreover, proportions of hTh1 cells were similar between Ctrl (48.1%) and PTCy-treated mice (52.1–70.9%) (*P*=0.30) (Figure 9I). Proportions of splenic hTc17 cells were near-absent and similar between Ctrl and PTCy-treated mice (0.1–0.8%) (*P*=0.63) (Figure 9J). Moreover, apart from one mouse in the PTCy 33 group, proportions of splenic hKIR^+^hCD8^+^ cells were relatively low and similar between control (0.54%) and PTCy-treated mice (0.75–2.3%) (*P*=0.70) (Figure 9K).

Splenic proportions of hUnconv T cells were similar between Ctrl (69.9%) and low-, mid- or high-dose PTCy-treated mice (61.8–64.6%) (*P*=0.44) (Figure 9L). Compared with Ctrl mice (6.7%), proportions of splenic hNK cells were similar in PTCy 10 (*P*=0.13) and PTCy 33 mice (*P*=0.19) but reduced by 89.4% in PTCy 25 mice (*P*=0.05), which approached statistical significance, noting a high degree of variation in hNK cell proportions in the control group (Figure 9M).

At day 28, the effects of PTCy at low-, mid- or high-doses on splenic hCD4^+^ T cells, hCD8^+^ T cells and hTregs expressing co-inhibitory receptors PD-1 and LAG3 were examined. PTCy at any dose did not affect splenic proportions of these subsets (Supplementary Figure S1).

### Low-dose PTCy reduces proportions of hepatic hUnconv T cells early in disease

**Figure 10:**
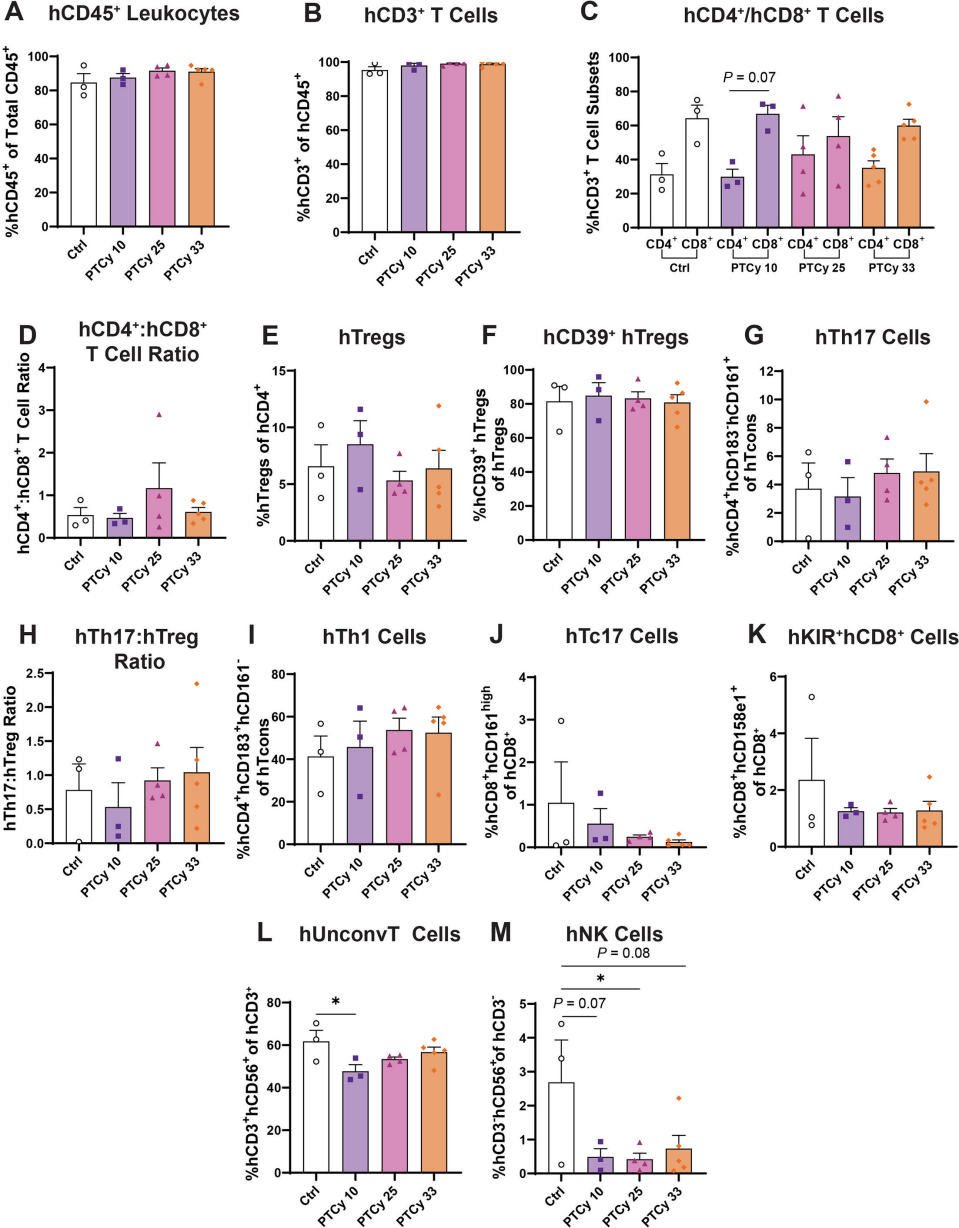
Low-dose PTCy reduces proportions of hepatic human UncovT cells early in disease. Livers from humanised mice were examined by flow cytometry using the gating strategy shown in Figure 3A to identify human (h) leukocyte subsets at day 28 or the ethical endpoint. (**A**) hCD45^+^ and mCD45^+^ leukocytes were gated before identifying (**B**) hCD3^+^ T cells, (**C**) hCD4^+^ and hCD8^+^ T cell subsets, (**D**) hCD4^+^:hCD8^+^ T cell ratios, (**E**) hTregs, (**F**) hCD39^+^ hTregs, (**G**) hTh17 cells, (**H**) hTh17:hTreg ratios, (**I**) hTh1 cells, (**J**) hTc17 cells, (**K**) hKIR^+^hCD8^+^ cells, (**L**) hUnconv T cells and (**M**) hNK cells. Data are presented as the mean ± standard error of the mean (*n*=3–5). Symbols represent individual mice. Data are from one experiment. Normality was assessed using a Shapiro–Wilk test and significance was tested using (**B, C, D, E, F, H, K, L, M**) one-way ANOVA or (**A, G, I, J**) Kruskal–Wallis tests. *P<0.05.

In the liver at day 28, PTCy at any dose did not affect engraftment of hCD45^+^ leukocytes, with each group showing similar proportions (87.5–91.6%) compared with Ctrl mice (84.6%) (*P*=0.42) (Figure 10A). Proportions of hCD3^+^ T cells were also similar between Ctrl (95.3%) and PTCy-treated mice (97.9–99.0%) (*P*=0.13) (Figure 10B).

In both Ctrl and PTCy-treated mice, proportions of hCD4^+^ T cells (29.9–43.0%) were lower than hCD8^+^ T cells (53.8–66.9%), with the difference between T cell subset proportions approaching statistical significance in PTCy 10 mice (*P*=0.07), but not other groups (Figure 10C). Nevertheless, hepatic hCD4^+^:hCD8^+^ T cell ratios were similar in all four groups (0.5–1.2) (*P*=0.48) (Figure 10D). Compared with Ctrl mice, hepatic proportions of hTregs were not impacted in PTCy 10, PTCy 25 or PTCy 33 mice, with similar proportions in all groups (5.3–8.5%) (*P*=0.63) (Figure 10E). Furthermore, proportions of hCD39^+^ Tregs were similar across all four groups (80.8–84.8%) (*P*=0.96) (Figure 10F).

Proportions of hepatic hTh17 cells were low and similar in Ctrl (3.7%) and PTCy-treated mice (3.2–4.9%) (*P*=0.86) (Figure 10G), as were hTh17:hTreg ratios (0.5–1.0) (*P*=0.75) (Figure 10H). Moreover, proportions of hepatic hTh1 cells were similar between Ctrl (41.3%) and PTCy-treated mice (45.7–53.7%) (*P*=0.63) (Figure 10I). Apart from one mouse in the Ctrl group, proportions of hepatic hTc17 cells were near-absent and similar between Ctrl (1.0%) and PTCy-treated mice (0.2–0.6%) (*P*=0.36) (Figure 10J) and proportions of hepatic hKIR^+^hCD8^+^ cells were also low and similar between Ctrl (2.4%) and PTCy-treated mice (1.2–1.3%) (*P*=0.56) (Figure 10K).

Compared with Ctrl mice (61.8%), hepatic proportions of hUnconv T cells were significantly reduced by 22% in PTCy 10 mice (*P*=0.03) but were similar in both PTCy 25 (53.4%) and PTCy 33 mice (56.7%) (*P*=0.17 and *P*=0.48, respectively) (Figure 10L). Moreover, proportions of hepatic hNK cells were reduced by 82% in PTCy 10 (*P*=0.07), by 84% in PTCy 25 (*P*=0.049) and by 73% in PTCy 33 mice (*P*=0.08) compared with Ctrl mice, with PTCy 10 and PTCy 33 groups approaching statistical significance, noting a high degree of variation in the Ctrl group (Figure 10M).

At day 28, the effects of PTCy at low, mid or high doses on hepatic hCD4^+^ T cells, hCD8^+^ T cells and hTregs expressing co-inhibitory receptors PD-1 and LAG3 were examined. Other than a trending 1.3-fold increase in hPD-1^+^hTregs in PTCy 25 mice, PTCy at any dose did not affect hepatic proportions of these subsets (Supplementary Figure S2).

## Discussion

This study investigated whether lower doses of PTCy (10 mg/kg and 25 mg/kg) are equally as efficacious as high-dose PTCy (33 mg/kg) in limiting GVHD and assessed the effects of these different PTCy doses on human immune cell subsets and T cell exhaustion at early and late disease time points in a preclinical humanised mouse model. Findings from this study revealed that low-dose PTCy (10 mg/kg), but not mid-dose PTCy (25 mg/kg), can mitigate clinical signs of GVHD with comparable efficacy to that of high-dose PTCy (33 mg/kg), with delayed disease onset and improved survival observed in both groups compared with the control group. Further, low-dose PTCy increased proportions of hPD-1^+^hCD4^+^ and hPD-1^+^hCD8^+^ T cells and increased proportions of hPD-1^+^ hTregs. Finally, this study revealed that high-dose PTCy, but not lower doses, increased proportions of exhausted hCD8^+^ T cells.

Treatment with low-dose PTCy reduced clinical signs of GVHD, with significantly delayed GVHD onset and increased survival. This was also observed in mice treated with high-dose PTCy, which our group has previously observed [44,47,67]. Markedly, 30% of mice treated with low-dose PTCy survived until the day 70 endpoint. This improved survival with low-dose PTCy also aligns with findings in an allogeneic mouse model [68], where 10 mg/kg PTCy administered on days 3 and 4 was sufficient to reduce GVHD. Conversely, in a recent study in humanised mice, treatment with mid-dose PTCy (25 mg/kg) did not abrogate GVHD development [69], aligning with outcomes in the current study. Therefore, it appears that the efficacy of PTCy is not directly proportional to the dose, with a bimodal response observed. However, given that low-dose PTCy was effective at prolonging survival, this reduced dose may be a better option to reduce toxicity seen with high-dose PTCy treatment.

Treatment with low-dose PTCy increased splenic proportions of hPD-1^+^hCD4^+^ and hPD-1^+^hCD8^+^ T cells, which were defined as activated T cells [28]. These results align with findings from a recent clinical study, where patients who received PTCy treatment had increased proportions of hPD-1^+^hCD4^+^ and hPD-1^+^hCD8^+^ T cells from 90 to 180 days post-transplantation [13]. The increased proportions of hPD-1^+^ T cells observed in humanised mice treated with low-dose PTCy may be related to the pharmacokinetics of cyclophosphamide in mice. A study in BALB/c mice found that a lower dose of 12.5 mg/kg cyclophosphamide led to faster passage of bioactive metabolites from the liver to the bloodstream compared with 25 mg/kg. Additionally, metabolites persisted longer in the bloodstream at a low dose [70]. As such, extended persistence of cyclophosphamide metabolites at a low dose may enhance CD4^+^ and CD8^+^ T cell activation, as evidenced by increased proportions of hPD-1^+^ T cell subsets. It is important to note that enhanced T cell activation does not necessarily equate to enhanced pathogenicity, and these subsets may represent stem-like progenitor exhausted T cells, a precursor to terminally exhausted T cells [71].

Low-dose PTCy, but not higher doses, increased proportions of hPD-1^+^hCD4^+^ and hPD-1^+^hCD8^+^ T cells, whereas high-dose PTCy, but not lower doses, increased proportions of hPD-1^+^hLAG3^+^hCD8^+^ T cells at the day 70 endpoint. The enrichment of hPD-1^+^hCD4^+^ and hPD-1^+^hCD8^+^ T cells with low-dose PTCy may reflect an activated or progenitor-exhausted population [72,73], whereas the increase in hPD-1^+^hLAG3^+^hCD8^+^ T cells with high-dose PTCy suggests a shift toward terminal exhaustion [30,74]. However, a limitation of this study is an absence of functional assays for PD-1^+^ subsets that are required to resolve these states. Future studies utilising proliferation and *ex vivo* restimulation assays with cytokine production analyses would determine whether these cells retain proliferative capacity and effector function or have progressed to terminal exhaustion [74–77]. Additionally, assessment of transcription factors such as TOX (or thymocyte selection-associated high mobility group box protein) and TCF-1 (or T cell factor-1) would distinguish progenitor versus terminal exhaustion states, respectively [78,79]. Nevertheless, activated CD8^+^ T cells contribute to GVHD pathogenesis in humanised mice [80,81], indicating that the exhausted CD8^+^ T cell phenotype observed with high-dose PTCy in this study may be contributing to reduced GVHD severity. Further, the increase in hPD-1^+^hLAG3^+^hCD8^+^ T cells does not appear to affect GVL immunity, as this dose of PTCy alone or in combination with other agents does not impair GVL responses in preclinical humanised mouse models [67,82].

PTCy increased hPD-1^+^hTregs at low, mid and high doses, while low-dose PTCy showed the greatest increase in the proportions of splenic hPD-1^+^hTregs. Though Tregs can modulate the immune response through the secretion of anti-inflammatory cytokines, Tregs can also inhibit an inflammatory response through cell–cell interactions through PD-1 and LAG3 with their canonical receptor ligands [83,84]. Furthermore, PD-1 signalling plays an important role in maintaining the suppressive capabilities of Tregs [85]. Overall, this suggests that Treg resilience is bolstered by PTCy, which may be a contributing factor to the mechanism by which PTCy reduces GVHD.

Mid-dose PTCy, but not low- or high-dose PTCy, reduced proportions of hLAG3^+^hCD4^+^ T cells and hLAG3^+^ hTregs. A previous study in an allogeneic mouse model, where T cells from LAG3-deficient mice were transplanted into BALB/c mice, found that the absence of LAG3 on CD4^+^ but not CD8^+^ T cells exacerbates GVHD [86]. This same study also observed that LAG3-deficient Tregs maintained the ability to suppress the proliferation of alloreactive T cells in a mixed lymphocyte reaction, indicating that LAG3 is not required for Treg function. As such, the reduced proportions of hLAG3^+^hCD4^+^ T cells rather than hLAG3^+^ hTregs could be a contributing factor to the lack of clinical efficacy of PTCy at 25 mg/kg in the current study.

Analysis of other immune cell subsets revealed several differences between mice treated with low-, mid- and high-dose PTCy. Mice treated with high-dose PTCy, but not low or mid doses, showed increased proportions of hTh1 cells and reduced proportions of hTh17 cells in the spleen, with a similar trend observed in the liver at the day 70 endpoint. Given the increase in hTh1 cells, which are widely considered the principal CD4^+^ T cell mediators of GVHD [54,87], these cells could be a contributing factor as to why mice treated with high-dose PTCy still succumb to the disease. Although the role of Th17 cells in GVHD is complex, this subset likely contributes to GVHD pathogenesis [88–90]. As such, the reduced proportions of hTh17 cells observed in high-dose PTCy-treated mice may contribute to the reduced GVHD severity observed in these mice. As this trend was not observed in mice treated with low-dose PTCy, these outcomes provide further evidence to suggest that PTCy may be reducing GVHD through different dose-dependent mechanisms.

Several studies have observed a role for NK cells in the pathogenesis of GVHD [91,92]. However, NK cells may also protect against GVHD. Several studies demonstrate that grafts containing higher numbers of NK cells and higher NK cell alloreactivity are associated with less severe GVHD [93–95]. In the current study, proportions of splenic and hepatic hNK cells were not reduced in mice treated with low-dose PTCy but were significantly reduced at day 28 in mice treated with mid-dose PTCy. The recovery of NK cells following alloHSCT has been identified as a risk marker for overall survival, with rates of relapse and non-relapse mortality significantly higher in patients with low NK cell counts of <50.5 cells/µl on day 28 post-transplant [96]. As such, the reduced proportions of hNK cells in mice treated with mid-dose PTCy early in disease may be a contributing factor as to why this treatment did not improve clinical GVHD outcomes in the current study.

Despite the delayed disease onset and prolonged survival of mice treated with low- or high-dose PTCy, there were no significant differences in histological GVHD in any of the target organs examined at day 70. The absence of histological improvements may be due to most mice having reached advanced disease by endpoint, potentially obscuring earlier treatment effects. However, there was a downward trend in liver GVHD at day 28 in mice treated with low- or high-dose PTCy. This trend is consistent with reduced liver GVHD in humanised mice treated with 33 mg/kg PTCy [44,47] and in allogeneic mice treated with 10 mg/kg PTCy [20]. Reasons for the differing magnitude of liver GVHD in humanised mice treated with high-dose PTCy remain unknown but may reflect donor differences or the heterogeneity of organ involvement in GVHD development.

A limitation of this study is that PTCy dosing in mice cannot be directly equated to clinical regimens due to species differences in body surface area, metabolism and pharmacokinetics. Given that NSG mice are more sensitive than humans to chemotherapy [97], 33 mg/kg PTCy represents an appropriate high dose in this model and was selected based on allogeneic mouse models of GVHD [24]. Our group has previously shown that this dose effectively reduces alloreactive donor T cell proliferation and GVHD [44] without compromising GVL immunity [82], supporting its value as a clinically relevant framework for dissecting the cellular mechanisms underlying PTCy efficacy in GVHD. Moreover, findings from the short-term model are constrained by the small sample size, and confirmation of these outcomes in larger cohorts is warranted.

The clinical implications of dose modification are considerable, given the well-established toxicities of cyclophosphamide at higher doses [98]. However, our study did not include direct measures of PTCy-associated toxicity. Future studies should incorporate toxicity assessments alongside efficacy, including (i) echocardiography with cardiac TUNEL (or terminal deoxynucleotidyl transferase dUTP nick end labeling) staining for cardiotoxicity [99,100]; (ii) daily urinalysis with bladder histology for urothelial injury [101]; (iii) liver function measurements of alanine aminotransferase, aspartate aminotransferase, alkaline phosphatase and total bilirubin for hepatotoxicity [102,103]; and (iv) renal function measurements of blood urea nitrogen and creatinine for nephrotoxicity [104]. To disentangle toxicity from GVHD-associated injury, these readouts should be evaluated in GVHD and non-GVHD cohorts receiving identical PTCy treatment regimens.

To conclude, this study aimed to determine the efficacy of lower doses of PTCy for GVHD attenuation and the subsequent effects on tissue damage and immune cell subsets at early and late time points in GVHD development using a preclinical humanised mouse model. Together, results from this study indicate that low-dose PTCy (10 mg/kg) reduces GVHD at a similar efficacy to high-dose PTCy (33 mg/kg). Moreover, the reduction in GVHD appears to be occurring through different dose-dependent mechanisms. Low-dose PTCy spared hCD8^+^ T cell exhaustion and increased proportions of hPD-1^+^hCD4^+^ T cells, hPD-1^+^hCD8^+^ T cells and PD-1^+^ hTregs. Conversely, high-dose PTCy led to a trending increase in hTh1 cell proportions and increased proportions of exhausted hCD8^+^ T cells. While an increase in exhausted hCD8^+^ T cells following treatment with high-dose PTCy may be beneficial for GVHD prevention, this may pose deleterious effects on GVL immunity. Therefore, treatment with low-dose PTCy, which increases T cell activation and avoids hCD8^+^ T cell exhaustion, may enhance GVL immunity, but further investigation into this notion is required. Furthermore, while low-dose PTCy attenuates GVHD, mice still succumb to disease. As such, future studies should investigate additional therapeutics to combine with low-dose PTCy to further improve clinical outcomes.

**Clinical Perspectives**

- Post-transplant cyclophosphamide (PTCy) is used to prevent graft-versus-host disease (GVHD) following allogeneic haematopoietic stem cell transplantation (alloHSCT), but GVHD still develops in 40-50% of patients, necessitating a deeper understanding of its cellular mechanisms of action.
- Findings from this study demonstrate that low-dose PTCy can reduce GVHD with similar efficacy as high-dose PTCy in a preclinical humanised mouse model, with disease attenuation associated with different dose-dependent cellular mechanisms.
- These findings suggest that a lower PTCy dose may improve outcomes for alloHSCT recipients.

## Supplementary material

Online supplementary material 1

## Data Availability

The data that support the findings of this study are available from the corresponding author upon reasonable request.

## Open Access

This article has been published open access under our Subscribe to Open programme, made possible through the support of our subscribing institutions, learn more here: https://portlandpress.com/pages/open_access_options_and_prices#conditional

## Abbreviations

ANOVA: analysis of variance
Ctrl: control
FSC-A: forward scatter area
FSC-H: forward scatter height
GVHD: graft-versus-host disease
GVL: graft-versus-leukaemia
IL: interleukin
KIR: killer cell immunoglobulin-like receptor
MST: median survival time
NIR: near infrared
NK: natural killer
NSG: NOD-scid-IL2Rγ^null^
PBS: phosphate buffered saline
PTCy: post-transplant cyclophosphamide
SSC-A: side scatter area
Tc: cytotoxic T
Tcon: conventional T
Th: T helper
Treg: regulatory T cell
Unconv: unconventional
alloHSCT: allogeneic haematopoietic stem cell transplantation
alloHSCT: allogeneic haematopoietic stem cell transplantation
h: human
hPBMC: human peripheral blood mononuclear cell
i.p.: intraperitoneally
