# Supplementary material for: Low- and high-dose post-transplant cyclophosphamide attenuates graft-versus-host disease with distinct effects on PD-1+ T cell subsets
Source: Clin Sci (Lond). 2025 Oct 28;139(21):1261–85. doi: 10.1042/CS20257272 (PMC12687457; doi:10.1042/CS20257272)
Supplement: Online supplementary material 1 [file cs-139-21-CS20257272-s001.docx]

**Low and High Dose Post-Transplant Cyclophosphamide Attenuate
Graft-versus-Host Disease with Distinct Effects on PD-1⁺ T Cell Subsets**

Chloe Sligar, Miles J. Jacobs, Amal Elhage, Ronald Sluyter, Debbie Watson

Molecular Horizons and School of Science, University of Wollongong, Wollongong, NSW, 2522, Australia.


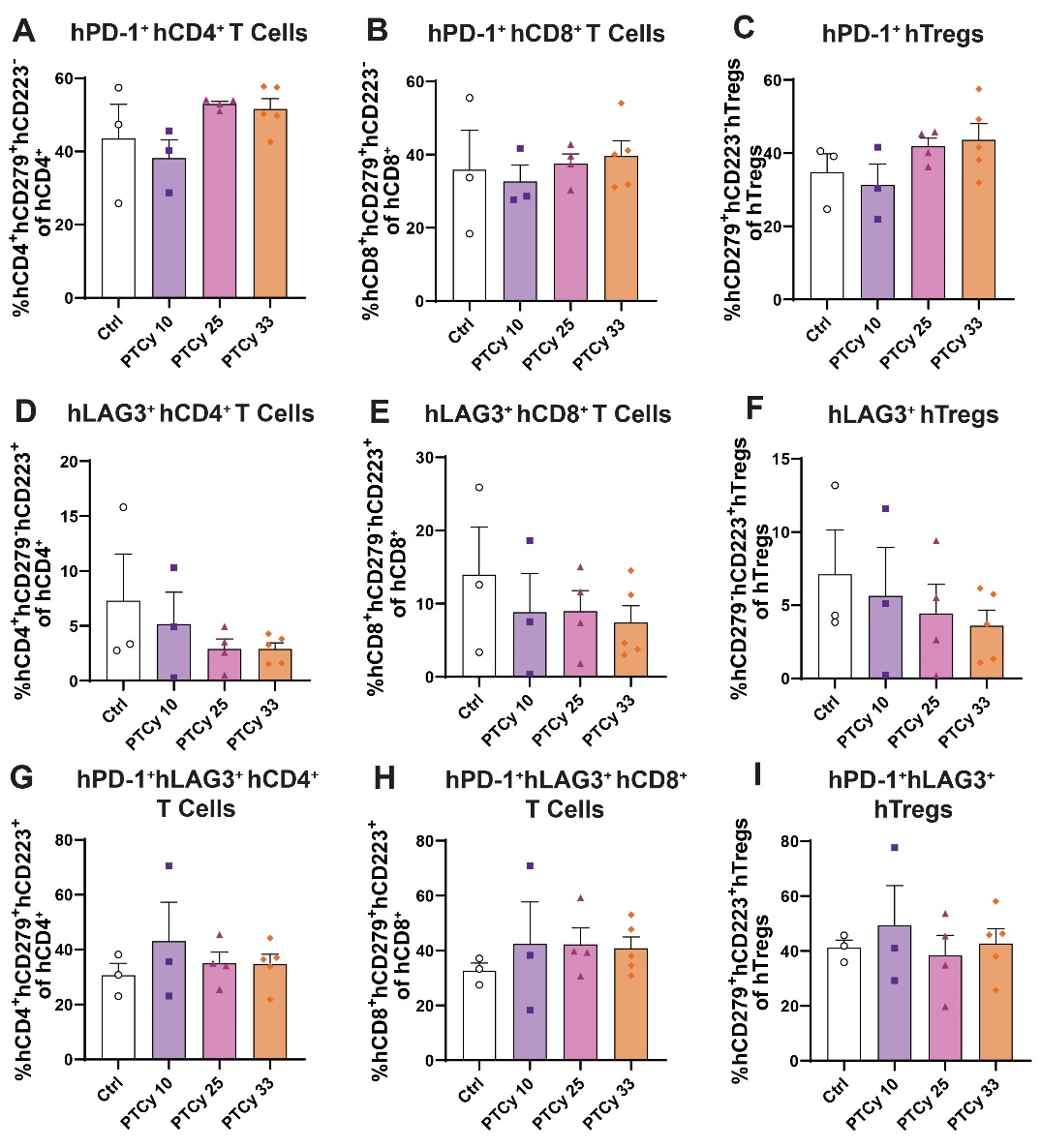
**Supplementary File**

#### **Supplementary Figure 1: PTCy at any dose does not impact the proportions of splenic human PD-1^+^, LAG3^+^ or PD-1^+^LAG3^+^ T cell subsets early in disease**. Spleens from humanised mice at day 28 or ethical endpoint were examined by flow cytometry. Proportions of human (h) CD45^+^ leukocytes, hCD3^+^ T cells, hCD4^+^ and hCD8^+^ T cell subsets and hTregs were identified using the gating strategy shown in Figure 3A before assessing hCD279^+^hCD223^-^ (hPD-1^+^), hCD279^-^hCD223^+^ (hLAG3^+^) and hCD279^+^hCD223^+^ (hPD-1^+^hLAG3^+^) subsets as shown in Figure 5A. Proportions of **(A)** hPD-1^+^hCD4^+^ T cells, **(B)** hPD-1^+^hCD8^+^ T cells, **(C)** hPD-1^+^hTregs, **(D)** hLAG3^+^hCD4^+^ T cells, **(E)** hLAG3^+^hCD8^+^ T cells, **(F)** hLAG3^+^hTregs, **(G)** hPD-1^+^hLAG3^+^hCD4^+^ T cells, **(H)** hPD-1^+^hLAG3^+^hCD8^+^ T cells and **(I)** hPD-1^+^hLAG3^+^hTregs were assessed. Data are presented as the mean ± standard error of the mean (n = 3-5). Data are from one experiment. Normality was assessed using a Shapiro-Wilk test and significance was tested using a **(A-I)** one-way ANOVA test.

####
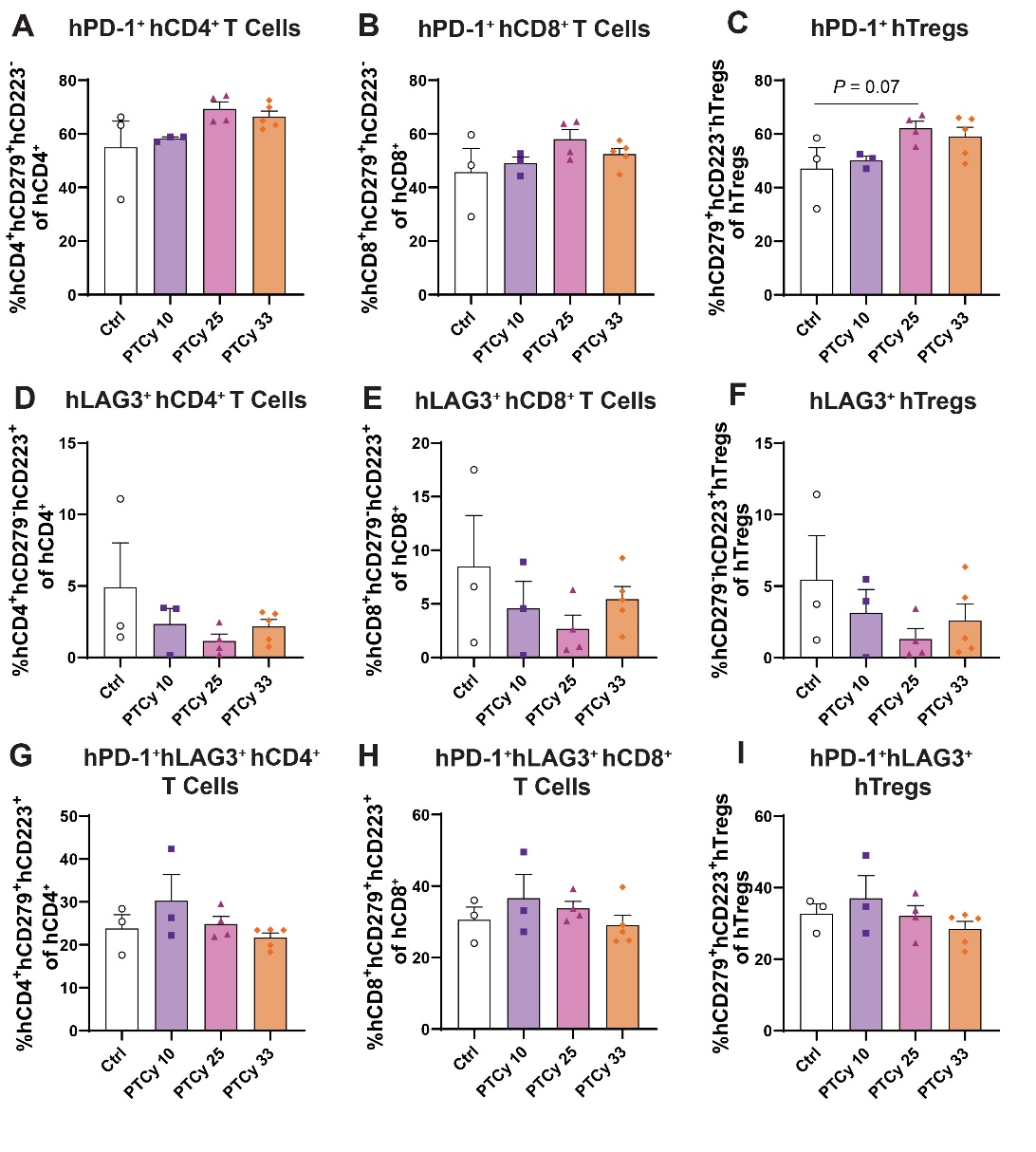
**Supplementary Figure 2: PTCy at any dose does not impact the proportions of hepatic human PD-1^+^, LAG3^+^ or PD-1^+^LAG3^+^ T cell subsets early in disease**. Livers from humanised mice at day 28 or ethical endpoint were examined by flow cytometry. Proportions of human (h) CD45^+^ leukocytes, hCD3^+^ T cells, hCD4^+^ and hCD8^+^ T cell subsets and hTregs were identified using the gating strategy shown in Figure 3A before assessing hCD279^+^hCD223^-^ (hPD-1^+^), hCD279^-^hCD223^+^ (hLAG3^+^) and hCD279^+^hCD223^+^ (hPD-1^+^hLAG3^+^) subsets as shown in Figure 5A. Proportions of **(A)** hPD-1^+^hCD4^+^ T cells, **(B)** hPD-1^+^hCD8^+^ T cells, **(C)** hPD-1^+^hTregs, **(D)** hLAG3^+^hCD4^+^ T cells, **(E)** hLAG3^+^hCD8^+^ T cells, **(F)** hLAG3^+^hTregs, **(G)** hPD-1^+^hLAG3^+^hCD4^+^ T cells, **(H)** hPD-1^+^hLAG3^+^hCD8^+^ T cells and **(I)** hPD-1^+^hLAG3^+^hTregs were assessed. Data are presented as the mean ± standard error of the mean (n = 3-5). Data are from one experiment. Normality was assessed using a Shapiro-Wilk test and significance was tested using **(B, C, E, F, G, H, I)** one-way ANOVA or **(A, D)** Kruskal-Wallis tests
